# Development of rice water-saving and drought resistance quantitative evaluation system of wide water ecological range based on quantitative gradient water control

**DOI:** 10.3389/fpls.2025.1548074

**Published:** 2025-02-25

**Authors:** Haiqi Kang, Muhammad Ahmad Hassan, Jiarong Kang, Yuehua Luo, Hong Zhang, Yongxuan Zeng, Guanfu Fu, Rongmin Qin, Deze Xu, Shimei Wang

**Affiliations:** ^1^ School of Tropical Agriculture and Forestry, Hainan University, Haikou, Hainan, China; ^2^ Rice Research Institute, Anhui Academy of Agricultural Sciences, Hefei, China; ^3^ Sichuan Academy of Agricultural Sciences, Chengdu, China; ^4^ Zhongzhi Shunguan Agricultural Group Co., Ltd, Nanning, Guangxi, China; ^5^ China National Rice Research Institute, Hangzhou, China; ^6^ Institute of Food Crops, Hubei Academy of Agricultural Sciences, Wuhan, Hubei, China

**Keywords:** *Oryza sativa*, drought resistance, water ecological range, water use efficiency, quantitative evaluation, complex drought resistance index

## Abstract

The drought resistance of rice is an indirect observational and complex trait whose phenotype is reflected in the response of directly observational traits to drought stress. To objectively and accurately evaluate the drought resistance of rice, soil moisture gradient quantification was designed as a general water index among different soil types. Through soil water control, water consumption calculation, yield test, trait examination, and statistical analysis, the relationship between quantitative water control treatment and rice yield drought resistance was studied to establish a quantitative and controllable evaluation system of rice drought resistance. Four kinds of gradients, namely, 100%, 80%, 60%, and 40% field moisture capacity, were designed in the experiment. Six tested rice varieties grew under the long-term water control treatment. Six varieties grew under four levels of field moisture capacity from transplanting and returning to green to maturity. The calculation of actual field moisture shows that the four design levels formed a significant gradient and reached a very significant difference. The gradient and quantitative water control (GQWC) significantly influenced tiller formation, grain yield, yield component traits, and water use efficiency. Under the designed GQWC treatment, the difference in yield drought resistance of tested rice varieties is reflected under wide water ecological amplitude. There was a significant difference between varieties and traits, and the relationship between traits and varieties was very significantly different under different GQWC levels. The differences in drought resistance among varieties differ due to various water gradients and direct observational traits. It is difficult to evaluate drought resistance accurately with a single gradient. Considering yield components and water use efficiency, it is the best choice for a comprehensive index with multi-gradient yield drought resistance. Based on the index mapping of gradient drought resistance and area calculations, 28 evaluation indices of drought resistance were calculated in parallel, and six indices with better evaluation effect were screened to solve the optimal comprehensive index, namely, the sum of drought resistance index under multi-gradient with multi-traits (MG_MT_DI_SUM), the sum of drought resistance index of yield under multi-gradient (MG_Y_DI_SUM), the product of total area under the curve of drought resistance index under multi-gradient with multi-traits (MG_MT_DI_TAUC_MUL), the drought resistance index of yield under the second gradient (SGII_Y_DI), the comprehensive value of membership function of the total area under the curve of drought resistance index with multi-gradient and multi-traits (MG_MT_DI_SUM), and the logarithm of total area under the curve of drought resistance index with multi-gradient and multi-traits (MG_MT_DI_TAUC_LOG). Among these indices, 100*MG_MT_DI_TAUC_LOG and 5*MG_Y_DI_SUM were the ideal evaluation indices, which could be used as the main indices for the comprehensive evaluation of rice drought resistance under the GQWC test.

## 1 Introduction

Upland rice (dry rice) was cultivated in China during the times of the Yaoshun Dynasty more than 4,000 years ago, and the cultivation techniques of upland rice were also recorded in Qi Min Yao Shu in the Northern Wei Dynasty (Wu et al., 2024). Dry rice is mainly distributed in dry and hilly land with normal summer rainfall but lacks irrigation conditions, or the drought-prone, high-altitude fields, and mountainous areas, as well as the low-lying land with spring drought and summer and autumn flooding; their yield is significantly lower than that of irrigated rice, generally 750–1,500 kg/hm^2^. It is still cultivated in mountainous and mid-level areas of Guangxi, Yunnan, Guizhou, and other provinces of China and arid and rain-fed regions of Henan and Hebei (Panda et al., 2021). With improved irrigation conditions and the spread of high-yield rice varieties, upland rice gradually decreased (Raza et al., 2023). However, in recent years, problems such as global climate change, lack of water resources, frequent seasonal drought and flood disasters, and the higher requirements of direct sowing rice encouraged the adaptation to change the mode of rice production, inspired by dry rice (Alexandrov et al., 2015); Luo et al. (2019) put forward the concept of water-saving and drought-resistant rice, believing that the development of water-saving and drought-resistant rice will be an important way for the sustainable development of rice production (Aroca, 2013). Water-saving and drought-resistant rice, derived from dry rice, is a type of cultivated rice that saves more than 50% of water in production than ordinary rice and can adapt to flood and drought production environments (Shultana et al., 2020). Therefore, dry rice is a good practice due to its good prospects for alleviating the water resources crisis, expanding the rice planting range, reducing greenhouse gas emissions and agricultural pollution, and ensuring food security (Anjum et al., 2011). Water-saving and drought-resistant rice breeding has become an important direction in the field of rice breeding, and its production has been widely demonstrated and promoted in major rice-producing areas such as Anhui, Hubei, Jiangxi, Hunan, Henan, Zhejiang, Jiangsu, Fujian, Guangxi, Hainan, Sichuan, Guizhou, and other places in China. Additionally, substantial promotional work has been carried out in Vietnam, Myanmar, Pakistan, Laos, Uganda, Ghana, and Madagascar in Africa (Anjum et al., 2017; Fahad et al., 2017).

Water-saving and drought-resilient rice include water conservation and drought-resistant characteristics, which are comprehensively reflected in its identifications (Fukagawa and Ziska, 2019). Water use efficiency (WUE) is the primary evaluation index of water-saving capacity, while drought resistance is more complex and has many evaluation methods (Luo, 2010). Currently reported methods of rice drought resistance identification mainly include 1) direct comparison method (Barik et al., 2019), 2) drought resistance grading evaluation method (Dixit et al., 2017), 3) total drought resistance evaluation method, and 4) mathematical analysis method (Gupta et al., 2020). The development period can also be divided into such methods as simulated drought stress at the germination stage (Hussain et al., 2018), phenotypic identification of drought response at the seedling stage (Kumar et al., 2017), and drought resistance identification at the whole growth stage (Luo et al., 2019). According to the identification of drought resistance of rice at different development stages, most reports were made on the germination stage and seedling stage, mainly using polyethylene glycol and mannitol to simulate drought stress and using germination rate, germination potential, germ length, main radicle length, coleoptile length, dried seed weight, germ dry weight, radicle dry weight, root–shoot ratio, and material transport rate as identification indices (Ali et al., 2017; Gopi and Manjula, 2018; Khanna et al., 2022). At the seedling stage, leaf rolling, dead leaves, and survival rate of repeated drought were used as drought resistance indicators, but the authors believed that the drought resistance indicators at the bud stage and seedling stage were not strongly correlated with the yield at the later stage (Cui et al., 2018).

Numerous and broader trait indicators are selected in rice drought resistance identification, mainly including botanical phenotypic indicators (leaf morphology, plant height, ear length, root morphology, etc.) (Basu et al., 2017; Bi et al., 2021); physiological and biochemical indices (leaf relative water content, chlorophyll content, leaf water potential, bound water content, stomatal resistance, plasma film permeability, tissue leaching conductivity, etc.) (He et al., 2024), stomatal changes (stomatal conductance, stomatal density, and stomatal length and width), osmotic regulatory substances (K^+^, Cl^-^, inorganic ions such as inorganic salts, etc.), and other inorganic ions; and organic solutes (such as proline and glycerol), protective enzymes, endogenous hormone changes (abscisic acid and polyamines, etc.), reactivation rate after drought stress (Augustine and Vierstra, 2018), yield, biological yield and their directly related agronomic traits (number of tillers per plant, effective panicle number per plant, the total number of grains per panicle, number of solid grains per panicle, thousand-grain weight, seed setting rate, seed density, and panicle weight), and other traits (Auler et al., 2017). Botanical phenotypic indices were studied most, and there were many indices of middle lobe and root morphology (Beznec et al., 2021). The root morphological indices (root number, root dry mass, root length, root thickness, root weight, root–shoot ratio, root power, root penetration, root pulling force, etc.) were relatively low in comparability, which was mainly based on the root pulling force identification method of the International Rice Research Institute (Bhatnagar et al., 2020). Some trait indicators include morphological and physiological indicators, and it is difficult for a single trait index to fully cover the drought resistance information of each line, so naturally, it cannot truly reflect the drought resistance of each line/variety (Chen et al., 2021).

Research on drought resistance of rice can refer to dryland crops such as wheat, corn, soybean, and cotton. Different evaluation algorithms have been reported in crop drought resistance research (Chengqi et al., 2024). It includes drought resistance coefficient (DC), drought resistance index (DRI), drought resistance index (DI) algorithm, sensitivity index (SI), drought damage index (ID), comprehensive drought resistance index, membership function analysis, comprehensive drought resistance D value, principal component analysis, gray correlation analysis, and other algorithms, as well as the combination of drought resistance index and membership function (Bailey-Serres et al., 2019), the principal component analysis combined with membership function analysis to construct drought resistance comprehensive evaluation value D-value algorithm (Chukwu et al., 2019), factor analysis and principal component analysis based on drought resistance coefficient to construct comprehensive evaluation index F algorithm, drought resistance coefficient and drought resistance index combined with gray correlation analysis comprehensive evaluation algorithm, and correlation analysis. The comprehensive evaluation algorithm of + membership function + comprehensive drought resistance coefficient + gray correlation + stepwise regression + cluster analysis, the comprehensive evaluation algorithm of principal component analysis + cluster analysis + correlation analysis + comprehensive D value with drought resistance coefficient as the index (Efendi et al., 2017), the membership function evaluation algorithm with drought resistance index as the index, the comprehensive drought resistance coefficient and drought resistance index algorithm, and the GGE biplot combined membership function comprehensive evaluation algorithm with drought resistance index as the trait index have gradually developed from a single index to a comprehensive analysis index (Fahad et al., 2017). The drought resistance coefficient is not stable from year to year. The drought resistance index can reflect the stable yield of varieties under different water conditions but cannot reflect water-saving characteristics (Pang et al., 2017). Drought resistance is closely related to water saving, and the study of water use efficiency is also a breakthrough for water saving and drought resistance (Choudhury et al., 2024). However, there are few reports on the identification index of water saving and drought resistance, combining drought resistance and water use efficiency (Martos et al., 2021).

In evaluating rice’s water saving and drought resistance, appropriate methods and indices should be used to distinguish the difference between water saving and drought resistance. Accurate identification of plant drought resistance has always been the most critical factor for the success of drought resistance research (Ortega‐Gaucin et al., 2021). Since the drought resistance of rice is a non-intuitive and complex trait, the effectiveness of “secondary traits” based on morphological, physiological, and biochemical indices in the indirect selection of water saving and drought resistance is problematic (Manickavelu et al., 2006). Studies have shown that the correlation between most phenotypes and physiological and biochemical traits of rice and yield in dry farming is less significant than that directly related to yield. Under drought stress conditions, rice grain yield has apparent advantages as a screening index for drought resistance, which can effectively reflect the drought resistance of rice varieties (Salehi-Lisar et al., 2012). Because the ultimate goal of rice drought resistance breeding is to breed high-yield and stable varieties under drought conditions, the yield should reflect the drought resistance of rice. In actual drought resistance breeding, direct selection of plant yield under drought stress is still a vital selection method for drought resistance breeding (Seleiman et al., 2021). For water-saving and drought-resistant rice breeding, it is necessary not only to minimize the yield loss after drought stress but also to make full use of limited agricultural water resources to produce as much rice as possible to achieve the water-saving and drought-resistant rice goal (Kumar et al., 2021). Therefore, one of the important directions of research on water-saving and drought-resistant rice is establishing a recognized evaluation index system of water-saving and drought-resistant rice by constructing comprehensive evaluation indices reflecting water-saving characteristics and drought resistance (Singh et al., 2016). For water-saving and drought-resistant rice, there is no ideal method index to identify water-saving and drought-resistant rice directly, and it is not easy to use specific quantitative indices to classify and compare water-saving and drought-resistant rice varieties (Kim et al., 2020).

Previously reported indices did not combine water saving and drought resistance (Han and Singh, 2023; Mukherjee et al., 2018); they were mainly limited to single-gradient and single/limited traits and could not widely reflect the comprehensive drought resistance under multi-gradient water conditions. The index reported in this paper is a logarithmic index that combines the drought resistance coefficients and drought resistance index of multi-traits under multi-gradients, which has never been reported in previous studies. Based on water quantity, we took yield as the primary trait in drought resistance evaluation, considered the coordinated change in water use efficiency, and designed a gradient quantitative water control experiment under a wide water ecological range. By drawing on the previous experience in crop drought resistance evaluation traits and algorithms, the author explores comprehensive evaluation indices for water saving and drought resistance of rice. A comprehensive index system for evaluating water-saving and drought-resistant rice breeding was sought to provide a useful evaluation technical index for the breeding and variety certification of water-saving and drought-resistant rice.

## 2 Materials and methods

### 2.1 Experimental design and growth conditions

**Figure 1:**
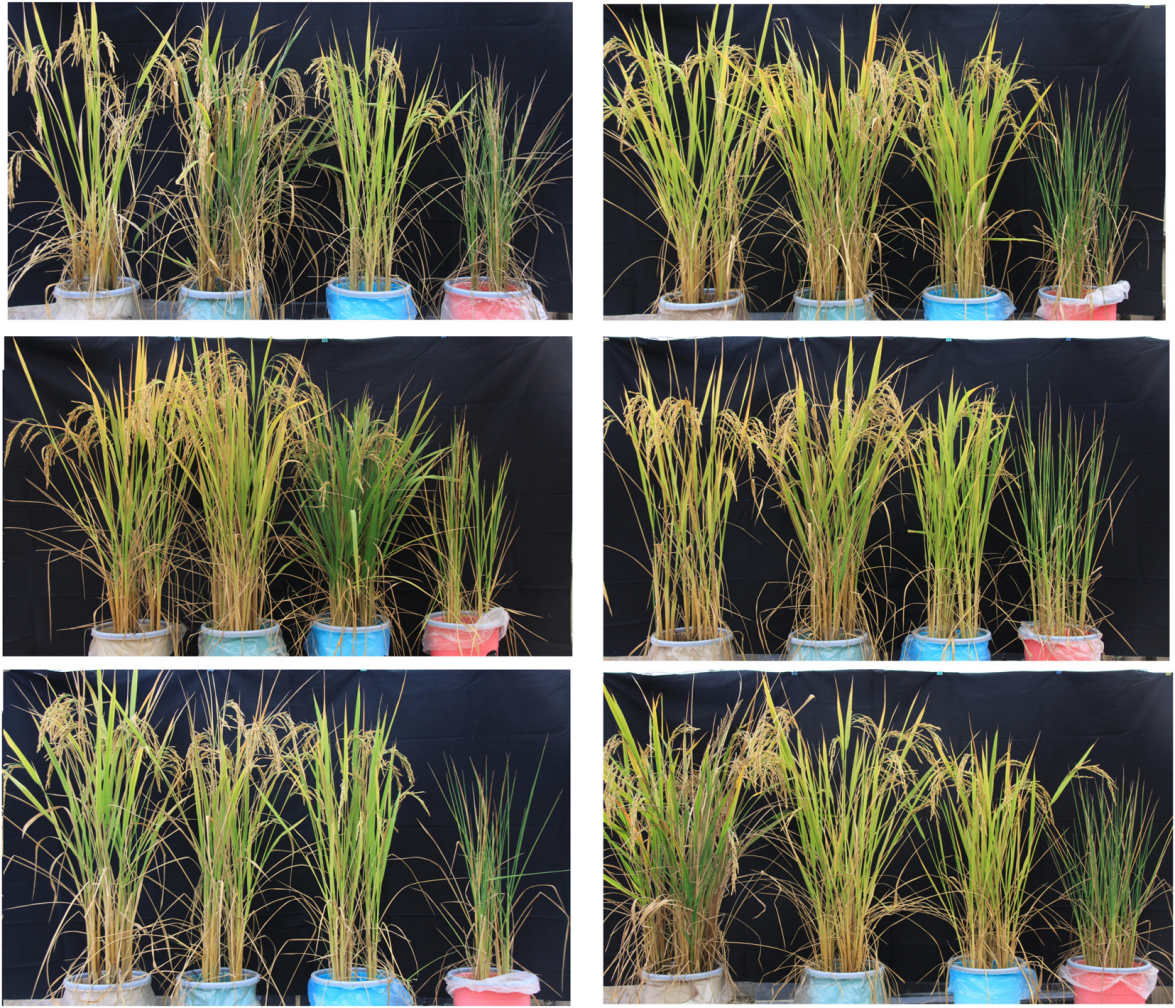
Impact of four different treatment levels of field capacity (left to right: 100%, 80%, 60%, and 40%) on four different varieties/lines [left to right in each row: IR64, Minghui 63 (MH63), II-32B, R17739-1, Bala, and Shuhui 527 (SH527)].

The pot experiment was carried out employing a completely randomized design (CRD) with a split arrangement, the field water capacity was used as the basis of soil water content gradient division, and the pot soil water content was quantified by the weighing method. Initially, each pot was filled with soil, and their uniformly constant weight (pot + soil) after adding soil was 7 kg. Later, on the day of transplantation, the measured quantity of water was added to each pot, and the weight of each pot reached 8.5 kg. Four water treatment levels were designed, namely, 100%, 80%, 60%, and 40% field capacity; morphological illustration of different treatment levels is exhibited in **Figure 1**. After the gradient quantification of water control, the pot was regularly weighed using an electronic balance (accuracy 0.001 kg). The total weight of each pot in the four treatments was always 9.5 kg, 7.82 kg, 7.14 kg, and 6.4 kg, and water was added when it was lower than this standard until harvest.

The diameter of each pot was 25 cm, and their depth was 30 cm. The experiment was carried out in a rain-proof greenhouse. The greenhouse was of 8-m displacement, and the length of the greenhouse arch was 10 m, which was covered with a water-proof plastic sheet, and its light transmittance was 95%. The two ends of the greenhouse were opened without any closure. Two fans were installed on the top of the arched greenhouse, which maintained the temperature in the greenhouse by internal and external air circulation. The two sides of the greenhouse had a 1.2-m-high arch without any plastic sheet and were ventilated all the time. Generally, on sunny days, the temperature inside the greenhouse was approximately 2°C–3°C higher than the ambient temperature outside.

### 2.2 Test materials

Six varieties were used in this experiment—IR64, Minghui 63 (MH63), II-32B, R17739-1, Bala, and Shuhui 527 (SH527)—among which IR64 is an internationally recognized water-sensitive variety, and Bala is a drought-resistant resource selected by our research group in the early phase of drought resistance identification, which is also supported by literature reports. MH63, Shuhui 527, and II-32B were the backbone parents of hybrid rice, and R17739-1 was a water-saving and drought-resistant rice restorer line independently bred by our research group. On April 15, the test materials were sown in the paddy field, and on May 30, they were transplanted into plastic pots. There were six pots in each treatment level, and three plants were planted (in a triangular shape) in each pot. All pots were placed in a rainproof greenhouse constructed by local workers. Pest and disease control was carried out according to local management practices.

### 2.3 Trait determination

During the experiment, the soil was weighed regularly, and the absolute water content of each pot was calculated according to the weight change. On June 15, the total number of tillers in each pot (three plants) was investigated once every 7 days, and the heading date was recorded. After harvest, the biological yield per plant (BYP), grain yield per plant (GYP), productive panicles per plant (PPP), total grains per panicle (TGP), filled grains per panicle (FGP), 1,000-grain weight (TGW), grain WUE (GWUE), and biological WUE (BWUE) were calculated based on water control data. The WUE was calculated by dividing the grain yield per plant by the total amount of water consumed per plant during the whole growth period. The formula for WUE was as follows:


$$\begin{array}{c} \text{WUE}=\text{Grain yield of a plant}÷\text{Total amount of water }\\ \text{consumed by a plant in the whole growth period}. \end{array}$$

Since the pot weight was continuously monitored during experimentation, it was possible to calculate the amount of water consumed per day.

### 2.4 Statistical analysis and drought resistance evaluation indices

EXCELL, DPS8.0, and other software were used to statistically analyze yield, structural properties, WUE, and other data. The BYP, GYP, PPP, TGP, FGP, TGW, GWUE, and BWUE of different varieties were analyzed by split-plot analysis.

According to the DC, DI, and membership function synthesis (MFSV) algorithms reported in the literature, the single gradient correlation index was first calculated (SG_Y). Later, the drought resistance coefficient of single gradient yield (SG_Y_DC), drought resistance index of single gradient yield (SG_Y_DI), sum of single gradient multi-trait DI (SG_MT_DI_SUM), and membership function synthesis of single gradient multi-trait DI (SG_MT_DI_MFSV) were calculated. The calculation formulas of DC, DI, MFSV, and LOG values for single traits of each variety under a single gradient are calculated as follows:


(1) $$\text{DC}=[{\text{X}}_{\text{dij}}/{\text{X}}_{\text{ck}}]\times 100\%$$

where ${\text{X}}_{\text{dij}}$ is the phenotypic value of the jth trait of the ith variety at the unsaturated field moisture capacity (FMC) level, and X_ck_ is the phenotypic value of the jth trait at the FMC level > 100%.

This research study used and improved the yield DI to apply all indices and drought stress gradients. The DI was calculated as follows:


(2) $$\text{DI}=\{\begin{array}{l} \frac{{\text{X}}_{\text{CK}}}{{\text{X}}_{\text{CK}}}*\frac{{\text{X}}_{\text{CK}}}{{\text{X}}_{\text{CK}}}[\text{Control}(\text{CK}):{\text{X}}_{\text{CK}}=\text{Trait value of the control treatment},\\ {\bar{\text{X}}}_{\text{CK}}=\text{Mean of control treatments}]\\ \frac{{\text{X}}_{\text{d}}}{{\text{X}}_{\text{CK}}}*\frac{{\text{X}}_{\text{d}}}{{\text{X}}_{\text{d}}}[\text{Under drought stress},\text{ all trait values were not }0:\\ \text{Xd}=\text{Trait values for stress treatment},\\ {\bar{\text{X}}}_{\text{d}}=\text{Mean of trait values for stress}]\\ \;0\;(\text{Under drought stress},\text{ all trait values were }0) \end{array}$$

The composite value of the membership function of the jth trait of the ith variety was calculated as follows.


(3) $$\begin{array}{c} {\text{MFSV}}_{\text{ij}}=\{\sum_{\text{j}=1}^{\text{m}}[\text{µ}(\text{Xj})\cdot \;[(\sqrt{\sum_{\text{i}=1}^{\text{n}}{\left(\text{Xij}-\text{X}.\text{j}\right)}^{2}})\\ /(\sqrt{\frac{1}{\text{N}}\sum_{\text{i}=1}^{\text{n}}{\left(\text{Xi}.-\text{µ}\right)}^{2}})]/\{\sum_{\text{j}=1}^{\text{m}}[(\sqrt{\sum_{\text{i}=1}^{\text{n}}{\left(\text{Xij}-\text{X}.\text{j}\right)}^{2}})/(\sqrt{\frac{1}{\text{N}}\sum_{\text{i}=1}^{\text{n}}{\left(\text{Xi}.-\text{µ}\right)}^{2}})]\}\} \end{array}$$

$$\text{X}(\mu )=(\text{X}-{\text{X}}_{\text{min}})/({\text{X}}_{\text{max}}-{\text{X}}_{\text{min}})$$

In addition, the total area under the curve (TAUC) composed of each DI point and the horizontal axis in the graph was calculated using the gradient quantification of yield constituent traits and the DI value of WUE under the condition of gradient quantification water control, and the TAUC_ij_ calculation formula of the jth trait of the ith variety was as follows:


(4) $${\text{TAUC}}_{\text{ij}}=0.1\times (1+2\times {\text{X}}_{i\text{jII}}+2\times {\text{X}}_{\text{ijIII}}+{\text{X}}_{\text{ijIV}})$$

On this basis, the sum, product, logarithm, and MFSV values of TAUC for each trait were calculated, which were used as the comprehensive effect values of the multi-gradient and multi-trait water ecology of rice varieties. On that basis, the multi-gradient yield drought resistance index (MG_Y_DI_SUM), multi-gradient and multi-trait drought resistance index (MG_MT_DI_SUM), multi-gradient and multi-trait drought resistance index membership function (MG_MT_DI_MFSV), multi-gradient and multi-trait TAUC sum (MG_MT_AUC_SUM), multi-gradient and multi-trait TAUC product (MG_MT_AUC_MUL), the logarithm of multi-gradient and multi-trait TAUC (MG_MT_AUC_LOG), and comprehensive value of multi-gradient and multi-trait TAUC membership function (MG_MT_AUC_MFSV) were calculated. Most of the previous studies only calculated the drought resistance coefficients and indices for a single trait with a single gradient, but this research study along with single-gradient and single-trait supplemented the multi-traits and multi-gradients; furthermore, it also included logarithmic values for each index. Hence, it is termed as a comprehensive and complex drought resistance index (CDRI).


$$\begin{array}{c} \text{MG\_MT}\_\text{TAUC}\_\text{SUM}\\ ={\text{TAUC}}_{\text{GYP}}+{\text{TAUC}}_{\text{PPP}}+{\text{TAUC}}_{\text{FGP}}+{\text{TAUC}}_{\text{TGW}}\\ +{\text{TAUC}}_{\text{GWUE}}+{\text{TAUC}}_{\text{BWUE}} \end{array}$$

$$\begin{array}{c} \text{MG}\_\text{MT}\_\text{TAUC}\_\text{MUL}\\ =[\frac{{\text{TAUC}}_{\text{GYP}}}{\left({\text{TAUC}}_{\text{PPP}}\times {\text{TAUC}}_{\text{FGP}}\times {\text{TAUC}}_{\text{TGW}}\right)}]\times (\frac{{\text{TAUC}}_{\text{BWUE}}}{{\text{TAUC}}_{\text{GWUE}}}) \end{array}$$

$$\begin{array}{c} \text{MG}\_\text{MT}\_\text{TAUC}\_\text{LOG}\\ =\log_{\text{TAUC}\_\text{GYP}}(\text{TAUC}\_\text{PPP}\times \text{TAUC}\_\text{FGP}\times \text{TAUC}\_\text{TGW})\\ \times \log_{\text{TAUC}\_\text{GWUE}}\text{TAUC}\_\text{BWUE} \end{array}$$

All 0 indicators were removed from the 28 indicators, and the R language corrplot package was used to make correlation plots for the remaining 26 indicators to visualize the correlation and significance between these indicators. The calculation formulas of DC, DI, MFSV, and LOG values for single traits of each variety under a single gradient have been calculated above. Here, i, j, and X in the calculation formula are expressed as follows: i denotes varieties [MH63, SH527, II-32B, Bala, R17739-1, and IR64], j denotes yield-related traits [PPP, FGP, TGW, GYP, GWUE, and BWUE], and X denotes gradient number [I, II, III, and IV]; furthermore, STDEV denotes standard deviation, CV denotes coefficient of variation, and Avr denotes the average. Other indices are calculated as follows:

DC value of GYP of the i variety with single gradient: SGX_Y_DC_i_ = $\left[{\text{X}}_{\text{dij}}/{\text{X}}_{\text{ck}}\right]$ × 100%.

Drought resistance index of the i variety GYP in single gradient: SGX_Y_DI_i_ = DI, as given in (ii).

The sum of drought resistance indices of multiple traits of the i varieties in a single gradient: SGX_MT_DI_SUM_i_ = $\sum_{j=1}^{6}SG\_X\_Y\_DI_{i,j}$

Membership function value of drought resistance index of multiple traits of the i varieties in single gradient: SGX_MT_DI_MFSV_i_ = $\sum_{j=1}^{6}DI\_MFSV_{i,j}$

DC value of GYP of the i variety with multiple gradients: MG_Y_DC_i_ = $\sum_{X=2}^{4}SG\_X\_Y\_DC_{i,X}$

The sum of DC values of multiple traits of i varieties with multiple gradients: MG_MT_DC_Sum_i_ = $\sum_{j=1}^{6}\sum_{X=2}^{4}DC_{i,j}$ The logarithm of multi-trait DC value of multi-gradient i varieties: MG_MT_DC_LOG_i_ = ${LOG}_{\sum_{X=2}^{4}SGX\_DC_{i,PPP}\times \sum_{X=2}^{4}DC_{i,FGP}\times \sum_{X=2}^{4}DC_{i,KGW}}^{\sum_{X=2}^{4}SGX\_DC_{i,GYP}}$ × ${LOG}_{\sum_{X=2}^{4}DC_{i,GWUE}}^{\sum_{X=2}^{4}DC_{i,BWUE}}$

Membership function value of multi-trait DC value of the i varieties with multiple gradients: MG_MT_DC_MFSV_i_ = MFSV_i,cDC_ × STDEV( $\sum_{X=2}^{4}DC_{i,j}$ )_j_/ $\sum_{j=1}^{6}$ STDEV( $\sum_{X=2}^{4}DC_{i,j}$ )_j_ The sum of the DC values of the total area under curves for multiple traits of the i varieties with multiple gradients: MG_MT_DC_TAUC_SUM_i_ = $\sum_{j=1}^{6}TAUC_{i,j}$

The multiplication product of the DC values of the total area under curves for multiple traits of the ith varieties with multiple gradients: MG_MT_DC_TAUC_MUL_i_ = $\sum_{j=1}^{6}DC\_TAUC_{i,j}$ Here, DC_TAUC_i,j_ is the total area under the curve the DC value curve of the jth trait of the ith variety.

Logarithmic values of the total area under the curve of DC values of the ith variety’s multiple traits in multiple gradients: MG_MT_DC_TAUC_LOG _I_ = ${LOG}_{DC\_TAUC_{i,}{{\;}_{PP}}_{P}\times DC\_TAUC_{i,}{{\;}_{FG}}_{P}\times DC\_TAUC_{i,}{{\;}_{K}}_{G}{\;}_{W}}^{DC\_TAUC_{i,GYP}}$ × ${LOG}_{DC\_TAUC_{i,G}{\;}_{WUE}}^{DC\_TAUC_{i,BWUE}}$

The multiplication product of the DC values of the total area under curves for multiple traits of the i (th) varieties with multiple gradients: MG_MT_DC_TAUC_MUL_i_ = [DC_TAUC_i, GYP_/(DC_TAUC_i, PPP_×DC_TAUC_i, FGP_ ×DC_TAUC_i, TGW_)]×(DC_TAUC_i, GWUE_ /DC_TAUC_iBWUE_), DC_TAUCi,j is the total area under the curve of DC value of the j (th) traits of the i (th) varieties

Membership function value of multi-trait DC value of the i varieties with multiple gradients.

Membership function of the total area under the curve of DC values of multiple traits of the ith varieties with multiple gradients: MG_MT_DC_TAUC_MFSV_i_ = $DC\_TAUC\_MFSV_{i,j}\times CV_{DC,j}/\sum_{j=1}^{6}CV_{DC,j}$ ,

CV_DC,j_ = STDEV(DC_TAUC_i,j1_, DC_TAUC_i,j2_, DC_TAUC_i,j3_, DC_TAUC_i,j4_, DC_TAUC_i,j5_, DC_TAUC_i,j6_)/Avr(DC_TAUC_i,j1_, DC_TAUC_i,j2_, DC_TAUC_i,j3_, DC_TAUC_i,j4_, DC_TAUC_i,j5_, DC_TAUC_i,j6_).

Here, STDEV is the standard deviation, Avr is the average, and j1, j2, …, j6 = [PPP, FGP, TGW, GYP, GWUE, BWUE].

The sum of DI values of the i variety GYP with multiple gradients: MG_Y_DI_SUM_i_ = $\sum_{X=1}^{4}DI_{i,GYP}$

The sum of DI values of multiple traits of the i varieties with multiple gradients: MG_MT_DI_SUM_i_ = $\sum_{X=1}^{4}\sum_{j=1}^{6}DI_{i,j}$

Membership function of DI value of multiple traits of the i varieties with multiple gradients: G_MT_DI_TAUC_MFSV_i_ = $DI\_TAUC\_MFSV_{i,j}\times CV_{DI,j}/\sum_{j=1}^{6}CV_{DI,j}$ ,

CV_DI,j_ = STDEV(DI_TAUC_i,j1_, DI_TAUC_i,j2_, DI_TAUC_i,j3_, DI_TAUC_i,j4_, DI_TAUC_i,j5_, DI_TAUC_i,j6_)/Avr(DI_TAUC_i,j1_, DI_TAUC_i,j2_, DI_TAUC_i,j3_, DI_TAUC_i,j4_, DI_TAUC_i,j5_, DI_TAUC_i,j6_).

The sum of total areas under the curve of the DI values of multiple traits of the i varieties with multiple gradients: MG_MT_DI_TAUC_SUM_i_ = $\sum_{j=1}^{6}DI\_TAUC_{i,j}$ ,

WHERE DI_TAUC_i,j_ is the area under the DI value curve of the jth trait of the ith variety.

The multiplication product of the total area under curves of the DI values of multiple traits of the i varieties with multiple gradients: MG_MT_DI_TAUC_MUL_i_ = [DI_TAUC_i, GYP_ /(DI_TAUC_i, PPP_×DI_TAUC_i, FGP_ ×DI_TAUC_i, TGW_ )]×(DI_TAUC_i, GWUE_ /DI_TAUC_iBWU_), DI_TAUCi,j is the total area under the curve of the DI values of the j (th) trait of the i (th) Variety

The logarithm of the total area under the curves of the DI values of multiple traits of the ith varieties with multiple gradients: MG_MT_DI_TAUC_LOG_i_ = ${LOG}_{DI\_TAUC_{i,}{{\;}_{PP}}_{P}\times DI\_TAUC_{i,}{{\;}_{FG}}_{P}\times DI\_TAUC_{i,}{{\;}_{K}}_{G}{\;}_{W}}^{DI\_TAUC_{i,GYP}}$ × ${LOG}_{DI\_TAUC_{i,G}{\;}_{WUE}}^{DI\_TAUC_{i,BWUE}}$

Membership function of the total area under the curve of the DI values of multiple traits of the ith varieties with multiple gradients: MG_MT_DI_TAUC_MFSV_i_ = MFSV_i, TAUC_ × STDEV( $\sum_{X=1}^{4}TAUC_{i,j}$ )_j_/ $\sum_{j=1}^{6}$ STDEV( $\sum_{X=1}^{4}TAUC_{i,j}$ )_j_.

Here, i, j, and X in the calculation formula are expressed as follows: i denotes varieties [MH63, SH527, II-32B, Bala, R17739-1, and IR64], j denotes yield-related traits [PPP, FGP, TGW, GYP, GWUE, and BWUE], and X denotes gradient number [I, II, III, and IV]; furthermore, STDEV denotes standard deviation, CV denotes coefficient of variation, and Avr denotes the average.

The DPS (18.10 version) software was used to carry out factor analysis on these 26 indicators. According to the factor scores, heat maps were made using the pheatmap package in R language, and Euclidean distance and shortest distance clustering analyses were carried out on the calculated evaluation indices. The evaluation index with good screening effect was selected as the evaluation index of drought resistance of rice varieties under the condition of gradient quantitative water control treatment and used for quantitative comparison of drought resistance of the tested rice varieties. These evaluation indices can be used well under the conditions of precise control of soil moisture, but they are difficult to apply in field conditions.

## 3 Results and analysis

### 3.1 Control of soil moisture content

**Figure 2:**
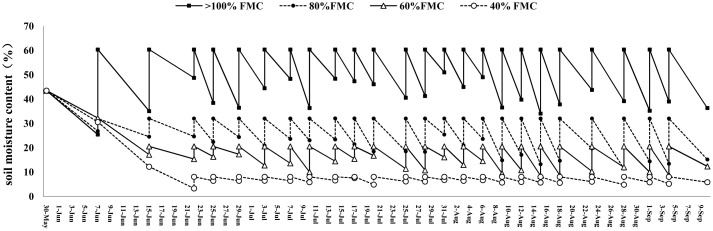
The gradient and quantitative control effect of soil water content.

During the period from regreening after transplanting to mature harvest, the soil absolute water content of four water treatment designs, namely, 100%, 80%, 60%, and 40% FMC, was regularly monitored. The results showed that the actual control of soil absolute water content between the four treatments achieved the expected effect, and the water content between the treatments reached a very significant difference (**Table 1**; **Figure 2**). The average absolute water content of the four designs was significantly different.

**Table 1:** The variation of absolute soil water content under four gradients and quantitative control.

Gradient levels	Average,%	Max.,%	Min.,%	SD	CV	Effective processing period (month/day)
100% field moisture capacity	48.972^**^	60.327	16.871	12.761	0.261	5/30–9/12
80% field moisture capacity	25.107^**^	33.216	8.409	8.018	0.319	6/7–9/12
60% field moisture capacity	15.861^**^	20.493	6.682	5.049	0.318	6/15–9/12
40% field moisture capacity	7.097^**^	10.685	3.235	1.284	0.181	6/22–9/12

CV, coefficient of variation.

** represent the significance probability levels at 0.01.

### 3.2 Effects of different water treatments on tillering

**Figure 3:**
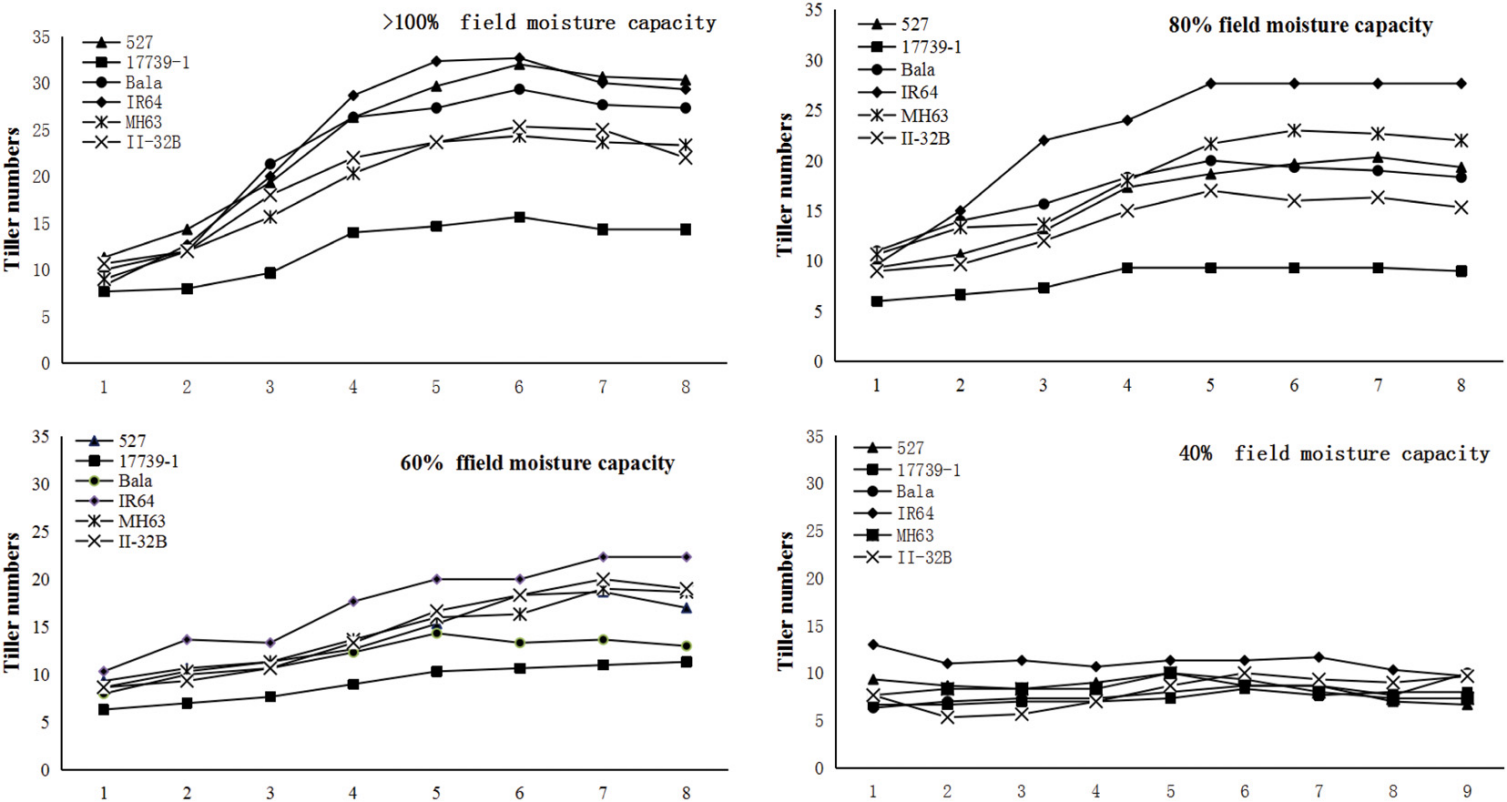
The influence of gradient and quantitative water control on tiller production.

Different water treatments had a very significant effect on the tillering formation of the six tested varieties. With the decrease in soil water content, the formation of tillering number per plant significantly changed (**Figure 3**). Under the condition of full irrigation, except that of R17739-1, the growth curve of tillering number per plant of the other five varieties was similar, which had the typical tillering characteristics. The number of tillers per plant of six varieties under 40% FMC did not increase or decrease significantly and remained in the basic seedling number state after transplantation. At 80% and 60% FMC levels, the growth curve of tillering number per plant was significantly different, but the relative ability of effective tillering was not consistent between the two levels. The relative ability of effective tillering, although significantly different, was inconsistent between the two levels.

### 3.3 Effects of multi-gradient drought stress on yield per plant and its constituent characteristics

From the perspective of single traits, the yield per plant and its component traits decreased significantly with the gradient decline of soil water content. Compared with those of 100% FMC, the yield and component traits of unsaturated FMC decreased significantly, and the lower the soil water content, the more significant the reduction (**Table 2**). The yield per plant, grain number per spike, GWUE, and BWUE significantly differed under the four water treatment levels. The yield per plant and grain number per spike under 80%, 60%, and 40% FMC were 63.10%, 15.23% 0%, and 77.13%, 27.15%, and 0%, respectively, under 100% FMC condition. The WUE of grain and biological yield were 72.66%, 29.69%, 0%, 104.83%, 96.28%, and 42.75% under 100% FMC condition. Therefore, these four water control treatments have obvious effects on the yield structure and WUE properties of the tested materials, and soil water content is the main reason for the change in properties.

**Table 2:** Average yield, yield component traits, and water use efficiency of tested varieties under various gradient levels of quantitative water control.

Gradient levels	GYP, g	Productive panicles/P (PPP)	Filled grains/panicle (FGP)	1,000-grain weight	Grains WUE (kg·m^−3^)	Biological WUE (kg·m^−3^)
100% field moisture capacity	11.95^a,A^	6.08^a,A^	84.73^a,A^	24.91^a,A^	1.28^a,A^	2.69^b,B^
80% field moisture capacity	7.54^b,B^	5.31^b,A^	65.35^b,B^	23.80^a,A^	0.93^b,B^	2.82^a,A^
60% field moisture capacity	1.82^c,C^	4.28^c,B^	23.00^c,C^	18.19^b,B^	0.38^c,C^	2.59 ^c,C^
40% field moisture capacity	0.00^d,D^	0.00^d,C^	0.00^d,D^	0.00^c,C^	0.00^d,D^	1.15^d,D^

Lowercase and capital letters represent significance probability levels at 0.05 and 0.01, respectively.

GYP, grain yield per plant; PPP, productive panicles per plant; FGP, filled grains per panicle; WUE, water use efficiency.

### 3.4 Yield composition characteristics and drought resistance index

**Figure 4:**
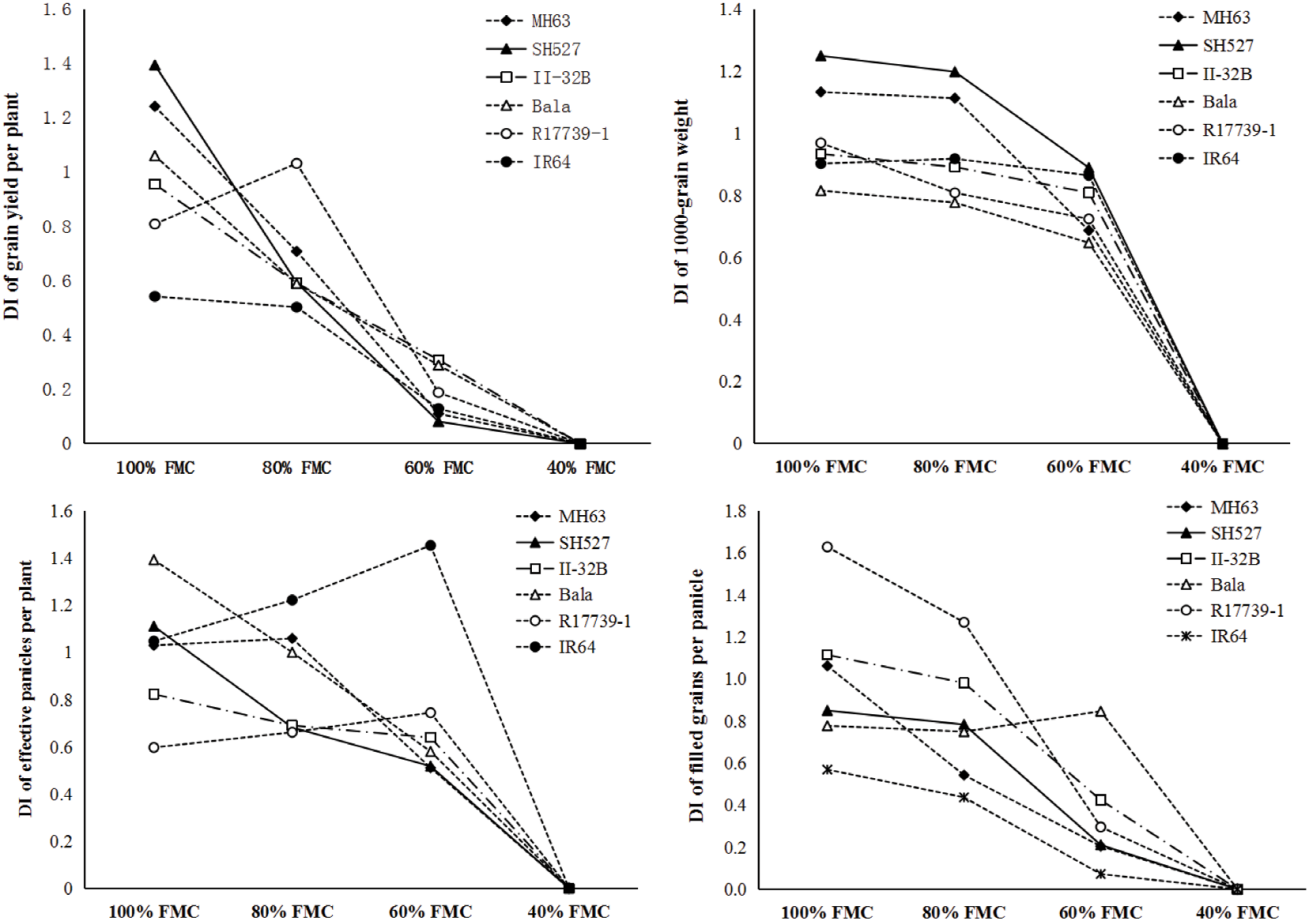
The drought resistance index (DI) of yield and its component traits on different controlling soil moisture levels.I, II, III, and IV represent four different treatment levels, respectively, 100%, 80%, 60%, and 40% field moisture capacity. Lowercase and capital letters represent significance probability levels at 0.05 and 0.01, respectively.

The drought resistance index of six varieties and their constituent traits at different water treatment levels is shown in **Figure 4**. Drought resistance indices of effective panicle per plant, number of grains per panicle, 1,000-grain weight, and yield per plant decreased with the increase of stress in most varieties, and the differences among materials also reduced with the increase of stress, but some varieties and traits were inconsistent with the changing trend. For example, the drought resistance index of effective panicle per plant of IR64 and R17739-1, the drought resistance index of grain number per panicle of Bala, and the drought resistance index of grain yield per plant of R17739-1 increased under 80% and 60% FMC than under 100% FMC. There was evident interaction among varieties, trait drought resistance index, and water gradient; there was no consistency between varieties and trait drought resistance index at different water gradient levels; there were different corresponding relationships between various traits and varieties. The difference in drought resistance between varieties was different due to the difference in water gradient and intuitive characteristics. The drought resistance index of six materials under 40% FMC was 0, the lowest value, which made it challenging to evaluate drought resistance. It can be seen that it was difficult to assess the consistency of drought resistance of materials with only one treatment level and a single characteristic.

### 3.5 Water use efficiency and drought resistance index

**Figure 5:**
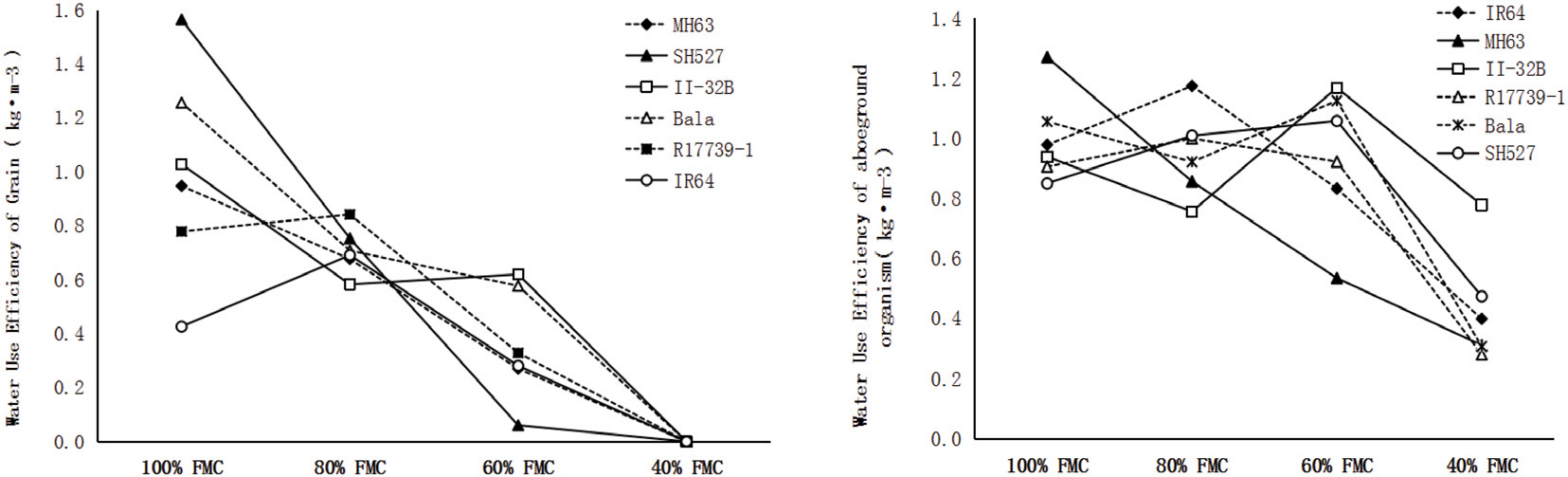
The drought resistance index (DI) of bio-aboveground water use efficiency (WUE) and grain WUE.

The GWUE and BWUE of the six materials showed significant differences in the four water treatment levels. GWUE and BWUE decreased with the gradient of soil water content. Under 80%, 60%, and 40% FMC gradient drought, GWUE was 70.9%, 35.6%, and 0% of 100% FMC, respectively; BWUE was 95.3%, 94.0%, and 42.5% of 100% FMC, respectively. The drought resistance index values of GWUE and BWUE also differed significantly under unsaturated soil moisture conditions (**Figure 5**). The average GWUE DI values of MH63, SH527, II-32B, Bala, R17739-1, and IR64 were 0.473, 0.594, 0.558, 0.635, 0.487, and 0.349, respectively. The average BWUE DI values were 0.846, 0.743, 0.911, 0.777, 0.852, and 0.848, respectively. It is difficult to reach a consistent conclusion on the drought resistance of varieties with the single gradient GWUE and BWUE drought resistance index values.

### 3.6 Comparative analysis of different evaluation indices of drought resistance

**Figure 6:**
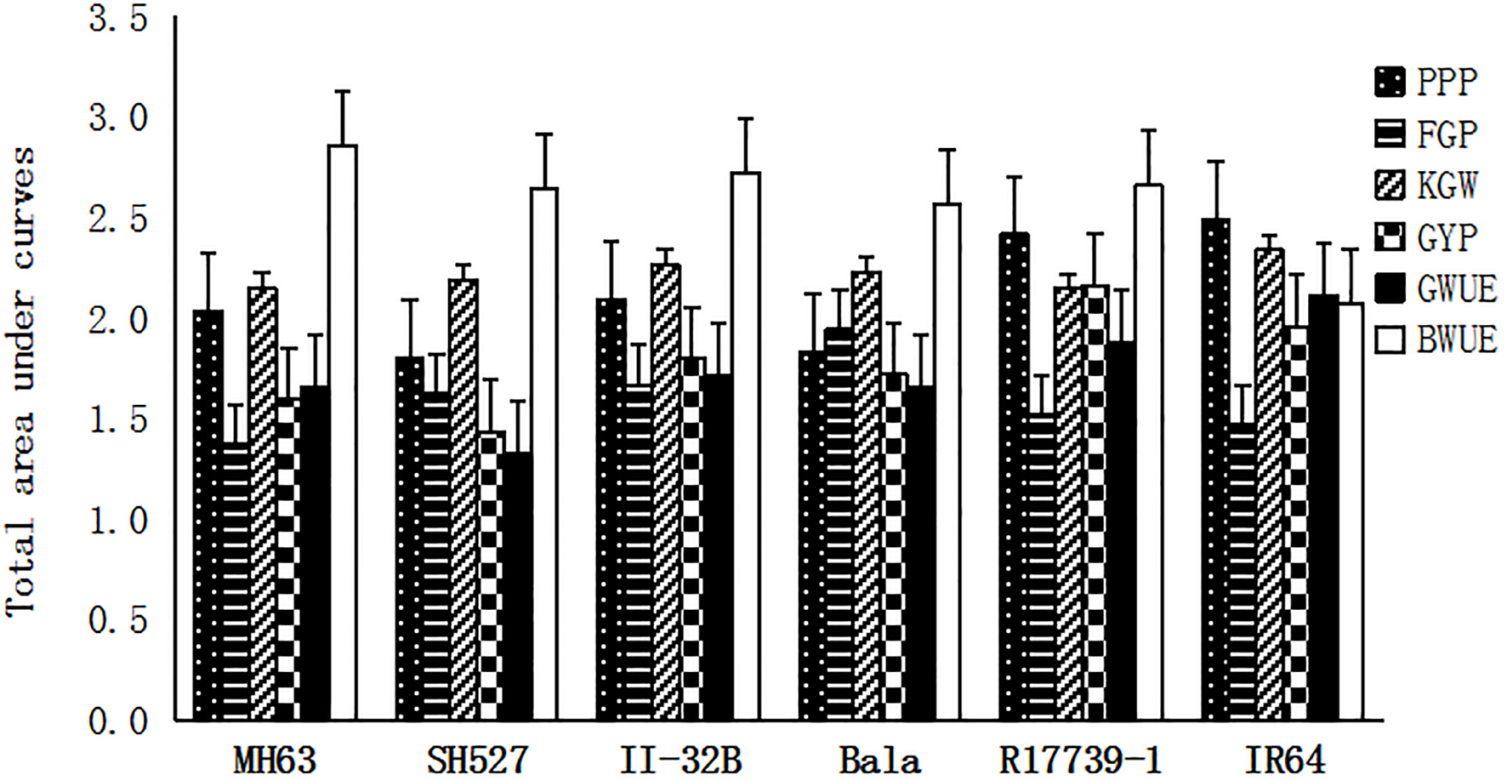
The total area of under curve of drought resistant coefficients (DRC) of yield trait and water use efficiency (WUE).

**Figure 7:**
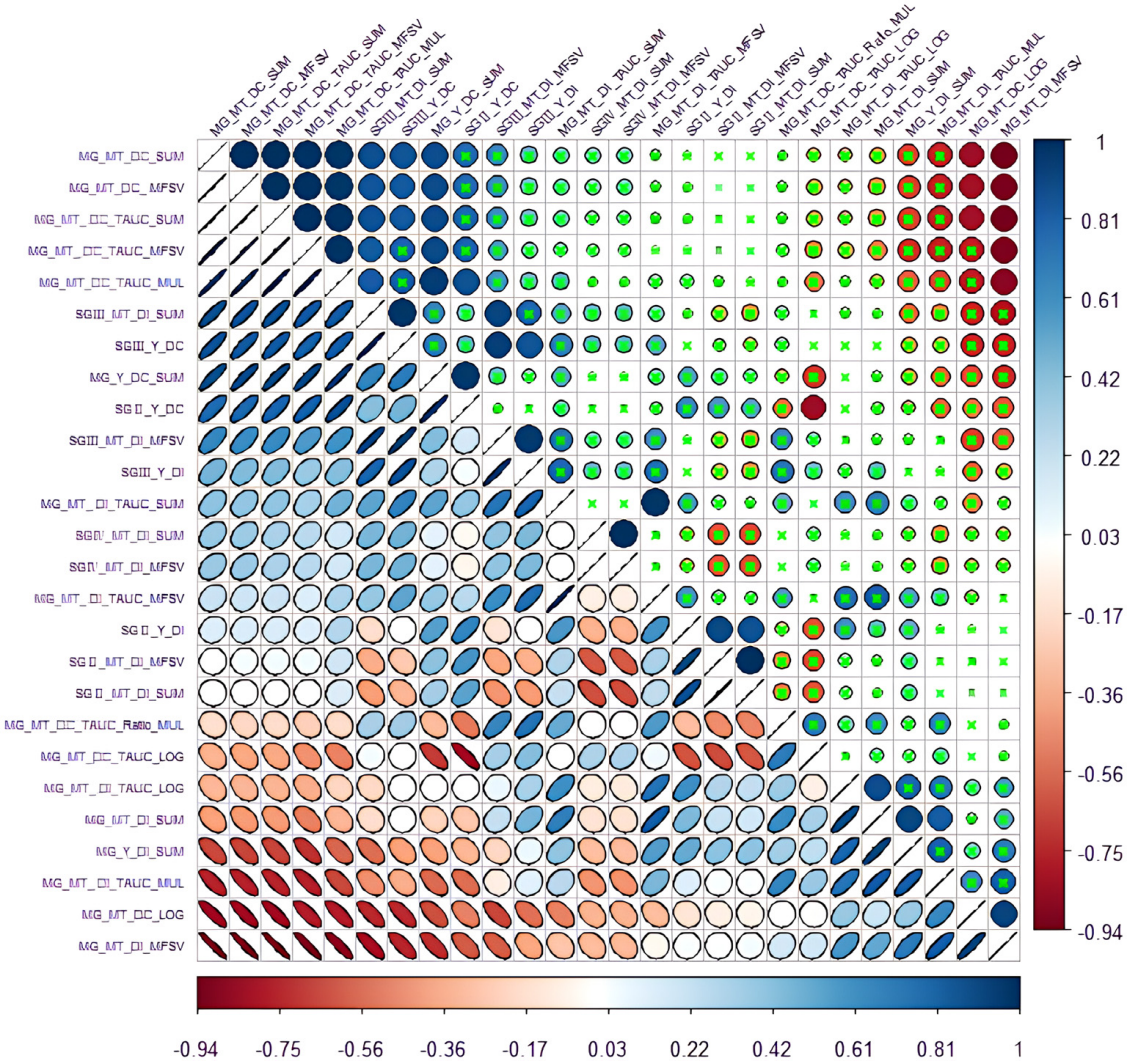
Correlation among 26 indicators (green “×” indicates that it is not significant at 0.05 level).

Based on **Figures 4** and **5**, the closed graph area (TAUC) composed of each DI point and the horizontal axis in the figure is regarded as the multi-gradient comprehensive drought resistance of the yield and composition traits of each variety (material) and water use efficiency. The results are shown in **Figure 6**. Furthermore, based on DC, DI, MFSV, gradient drought resistance index mapping, and area algorithm, 28 drought resistance evaluation values were calculated. Their results are shown in **Tables 3A–C**. Correlation analysis (**Figure 7**) showed that the logarithm (MG_MT_DC_LOG) of the multi-gradient and multi-trait drought resistance coefficients and the composite value (MG_MT_DI_MFSV) of the membership function of the multi-gradient and multi-trait drought resistance index had a significant negative correlation with the five multi-gradient and multi-trait drought resistance coefficients, including the multi-gradient and multi-trait drought resistance coefficient and (MG_MT_DC_SUM). The drought resistance coefficient (SGII_Y_DC) of gradient 2 yield was negatively correlated with the logarithm of the total area (MG_MT_DC_TAUC_LOG) of the multi-gradient and multi-trait drought resistance coefficient diagram. There was a significant correlation between some gradient three (SGIII) indices and the drought resistance coefficient (MG_MT_DC) index of multi-gradient and multi-trait. There was a significant correlation between the drought resistance index indicators of partial gradient two (SGII). There was a significant correlation between the drought resistance index and (MG_MT_DI_SUM) of multi-gradient and multi-trait drought resistance coefficient plots of total area logarithm (MG_MT_DC_TAUC_LOG), the drought resistance index of multi-gradient yield and (MG_Y_DI_SUM), and the drought resistance index and (MG_MT_DI_SUM) of multi-gradient and multi-trait drought resistance index. It can be seen that gradient 2 and gradient 3 significantly affected the multi-gradient index.

**Table 3A:** The index calculation of different evaluation methods for rice drought tolerance (1).

Evaluation method Varieties	Drought resistance coefficient of single-gradient yield traits (SG_Y_DC)			Drought resistance index of single-gradient yield traits (SG_Y_DI)			Single-gradient multi-trait DI (SG_MT_DI_SUM)			Composite value of DI affiliation function of single-gradient multi-traits (SG_MT_DI_MFSV)		
	SGII_Y_DC	SGIII_Y_DC	SGIV_Y_DC	SGII_Y_DI	SGIII_Y_DI	SGIV_Y_DI	SGII_MT_DI_SUM	SGIII_MT_DI_SUM	SGIV_MT_DI_SUM	SGII_MT_DI_MFSV	SGIII_MT_DI_MFSV	SGIV_MT_DI_MFSV
MH63	0.607	0.122	0	0.708	0.11	0	5.273	2.614	0.399	0.497	0.203	0.239
SH527	0.524	0.099	0	0.593	0.081	0	4.865	2.293	0.311	0.369	0.099	0.062
II-32B	0.632	0.233	0	0.591	0.309	0	4.492	3.968	0.778	0.259	0.676	1
Bala	0.599	0.214	0	0.589	0.289	0	4.822	3.862	0.28	0.361	0.724	0
R17739-1	0.908	0.198	0	1.032	0.188	0	5.533	3.406	0.307	0.62	0.416	0.053
IR64	0.774	0.2	0	0.502	0.129	0	4.779	3.857	0.474	0.341	0.415	0.389

I, II, III, and IV represent gradients 1, 2, 3, and 4, respectively. Y denotes yield, SG denotes single-gradient, MG denotes multiple gradients, MT denotes multiple traits, SUM denotes sum of added values, MUL denotes multiplication product of given values, TAUC denotes total area under the curve, LOG denotes the calculated logarithmic value, MFSV denotes membership function value, DC denotes drought resistance coefficients, and DI denotes drought resistance index. Calculation formulas of DC, DI, LOG, and MFSV are given in Subsection 2.4. Abbreviated symbols of calculated indices are as follows: i) SG_Y_DC denotes drought resistance coefficient of yield under the single-gradient n (1, 2, 3, and 4), ii) SG_Y_DI denotes drought resistance index of yield under the single-gradient n (1, 2, 3, and 4), iii) SG_MT_DI_SUM denotes the sum of drought resistance index of multiple yield traits under the single gradient n (1, 2, 3, and 4), and iv) SG_MT_DI_MFSV denotes membership function value of drought resistance index of multiple yield traits under single gradient n (1, 2, 3, and 4).

**Table 3B:** The index calculation of different evaluation methods for rice drought tolerance (2).

Evaluation method Variety	Multi-gradient yield drought resistance coefficients (MG_Y_DC)	The sum of drought resistance coefficient of multi-gradient and multi-trait (MG_MT_DC_SUM)	The logarithm of multi-gradient and multi-trait drought-resistant lines (MG_MT_DC_LOG)	Composite value of affiliation function of drought resistance coefficient of multi-gradient and multi-trait (MG_MT_DC_MFSV)	The sum of the total area of the multi-gradient and multi-trait drought resistance coefficient (MG_MT_DC_TAUC_SUM)	Multi-gradient and multi-trait drought resistance coefficient multiply the total area of each other (MG_MT_DC_TAUC_MUL)	The logarithm of the total area of the multi-gradient and multi-trait drought resistance coefficient plot (MG_MT_DC_TAUC_LOG)	Multi-gradient and multi-trait drought resistance coefficient plots, the product of the total area ratio (MG_MT_DC_TAUC_MUL)
MH63	0.73	8.128	0.004	0.355	2.185	0.002	0.1263	0.879
SH527	0.62	7.152	0.366	0.082	1.999	0.001	0.1559	0.824
II-32B	0.87	8.957	−0.006	0.588	2.333	0.003	0.1738	0.908
Bala	0.81	8.647	0.001	0.489	2.294	0.003	0.2212	0.928
R17739-1	1.11	9.136	0.021	0.651	2.393	0.004	0.1292	0.657
IR64	0.97	9.643	−0.011	0.795	2.527	0.004	0.1164	0.748

Y denotes yield, MG denotes multiple gradients, MT denotes multiple traits, SUM denotes sum of added values, MUL denotes multiplication product of given values, TAUC denotes total area under the curve, LOG denotes the calculated logarithmic value, MFSV denotes membership function value, DC denotes drought resistance coefficients, and DI denotes drought resistance index. Calculation formulas of DC, DI, LOG, and MFSV are given in Subsection 2.4. Abbreviated symbols of calculated indices are as follows: i) MG_Y_DC denotes drought resistance coefficient of yield under the multiple gradients n (1, 2, 3, and 4), ii) MG_MT_DC_SUM denotes the sum of drought resistance coefficients of multiple yield traits under the multiple gradients n (1, 2, 3, and 4), iii) MG_MT_DC_LOG denotes the logarithmic value of drought resistance coefficients of multiple yield traits under the multiple gradients n (1, 2, 3, and 4), iv) MG_MT_DC_MFSV denotes membership function value of drought resistance coefficients of multiple yield traits under multiple gradients n (1, 2, 3, and 4), v) MG_MT_DC_TAUC_SUM denotes sum of the values of total area under the curve formed by the number of varieties’ drought resistance coefficients of multiple yield traits under multiple gradients n (1, 2, 3, and 4), vi) MG_MT_DC_TAUC_MUL denotes multiplication product values of total area under the curve formed by the number of varieties’ drought resistance coefficients of multiple yield traits under multiple gradients n (1, 2, 3, and 4), and vii) MG_MT_DC_TAUC_LOG denotes the logarithmic values of total area under the curve formed by the number of varieties’ drought resistance coefficients of multiple yield traits under multiple gradients n (1, 2, 3, and 4).

**Table 3C:** The index calculation of different evaluation methods for rice drought tolerance (3).

Evaluation method Variety	The comprehensive value of the affiliation function of the total area of the multi-gradient and multi-trait drought resistance coefficient graph (MG_MT_DC_TAUC_MFSV)	Multi-gradient yield drought resistance index (MG_Y_DI_SUM)	Multi-gradient multi-trait drought resistance index (MG_MT_DI_SUM)	Composite value of membership function of drought resistance index with multiple gradients and multiple traits (MG_MT_DI_MFSV)	The sum of the total area of the multi-gradient and multi-trait drought resistance index plots (MG_MT_DI_TAUC_SUM)	The logarithm of the total area of the multi-gradient multi-trait drought resistance index graph (MG_MT_DI_TAUC_LOG)	The product of the total area of the multi-gradient and multi-trait drought resistance index plot (MG_MT_DI_TAUC_MUL)	The comprehensive value of the membership function of the total area of the multi-gradient and multi-trait drought resistance graph (MG_MT_DI_TAUC_MFSV)
MH63	0.309	2.059	14.677	1.309	2.256	1.177	0.044	0.437
SH527	0.089	2.068	14.907	1.478	2.206	1.637	0.088	0.414
II-32B	0.531	1.855	15.03	1.283	2.349	1.201	0.083	0.54
Bala	0.484	1.938	15.17	1.279	2.385	1.532	0.068	0.592
R17739-1	0.656	2.028	15.082	1.284	2.402	1.261	0.104	0.609
IR64	0.803	1.173	13.445	1.187	2.208	0.76	0.009	0.347

Y denotes yield, MG denotes multiple gradients, MT denotes multiple traits, SUM denotes sum of added values, MUL denotes multiplication product of given values, TAUC denotes total area under the curve, LOG denotes the calculated logarithmic value, MFSV denotes membership function value, DC denotes drought resistance coefficients, and DI denotes drought resistance index. Calculation formulas of DC, DI, LOG, and MFSV are given in Subsection 2.4. Abbreviated symbols of calculated indices are as follows: i) MG_MT_DC_TAUC_MFSV denotes the member function values of total area under the curve formed by the number of varieties’ drought resistance coefficients of multiple yield traits under multiple gradients n (1, 2, 3, and 4), ii) MG_Y_DI_SUM denotes the sum of the values of drought resistance index of yield under the multiple gradients n (1, 2, 3, and 4), iii) MG_MT_DI_SUM denotes the sum of the values of the drought resistance index of multiple yield traits under the multiple gradients n (1, 2, 3, and 4), iv) MG_MT_DI_MFSV denotes membership function values of drought resistance index of multiple yield traits under multiple gradients n (1, 2, 3, and 4), v) MG_MT_DI_TAUC_SUM denotes sum of the values of total area under the curve formed by the number of varieties’ drought resistance index of multiple yield traits under multiple gradients n (1, 2, 3, and 4), vi) MG_MT_DI_TAUC_LOG denotes the logarithmic values of total area under the curve formed by the number of varieties’ drought resistance index of multiple yield traits under multiple gradients n (1, 2, 3, and 4), vii) MG_MT_DI_TAUC_MUL denotes multiplication product values of total area under the curve formed by the number of varieties’ drought resistance index of multiple yield traits under multiple gradients n (1, 2, 3, and 4), and vii) MG_MT_DI_TAUC_MFSV denotes the member function values of total area under the curve formed by the number of varieties’ drought resistance index of multiple yield traits under multiple gradients n (1, 2, 3, and 4).

**Figure 8:**
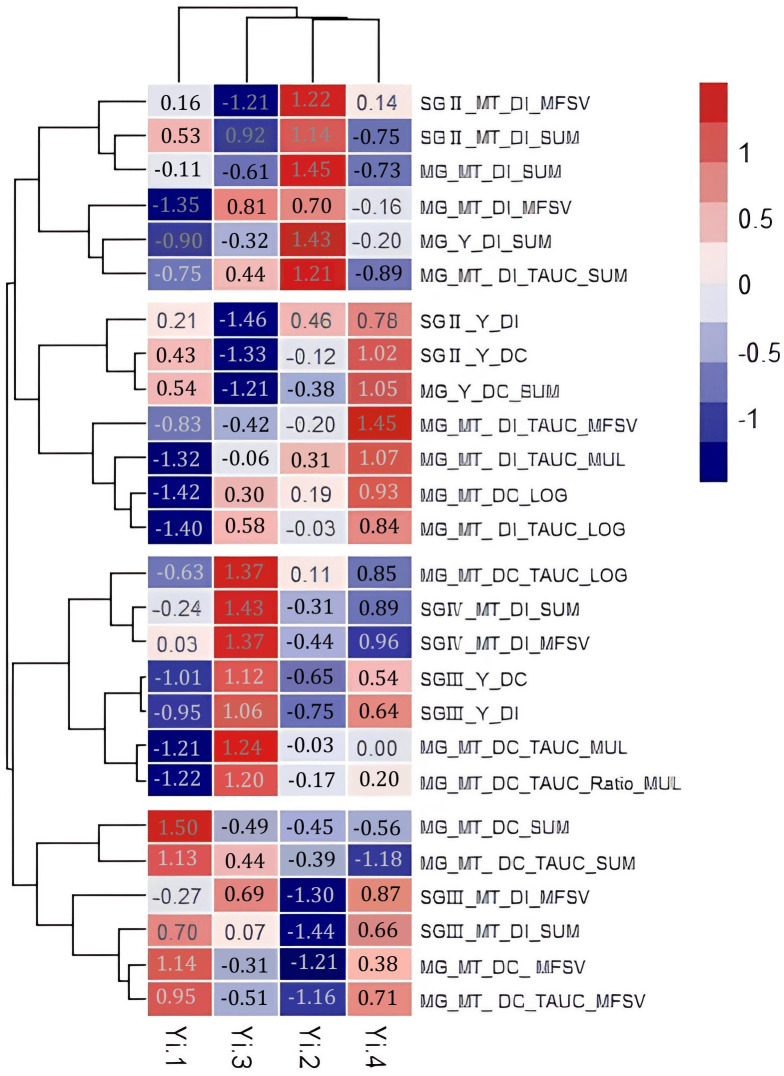
Factor score clustering of factor analysis of different evaluation indices.

**Figure 9:**
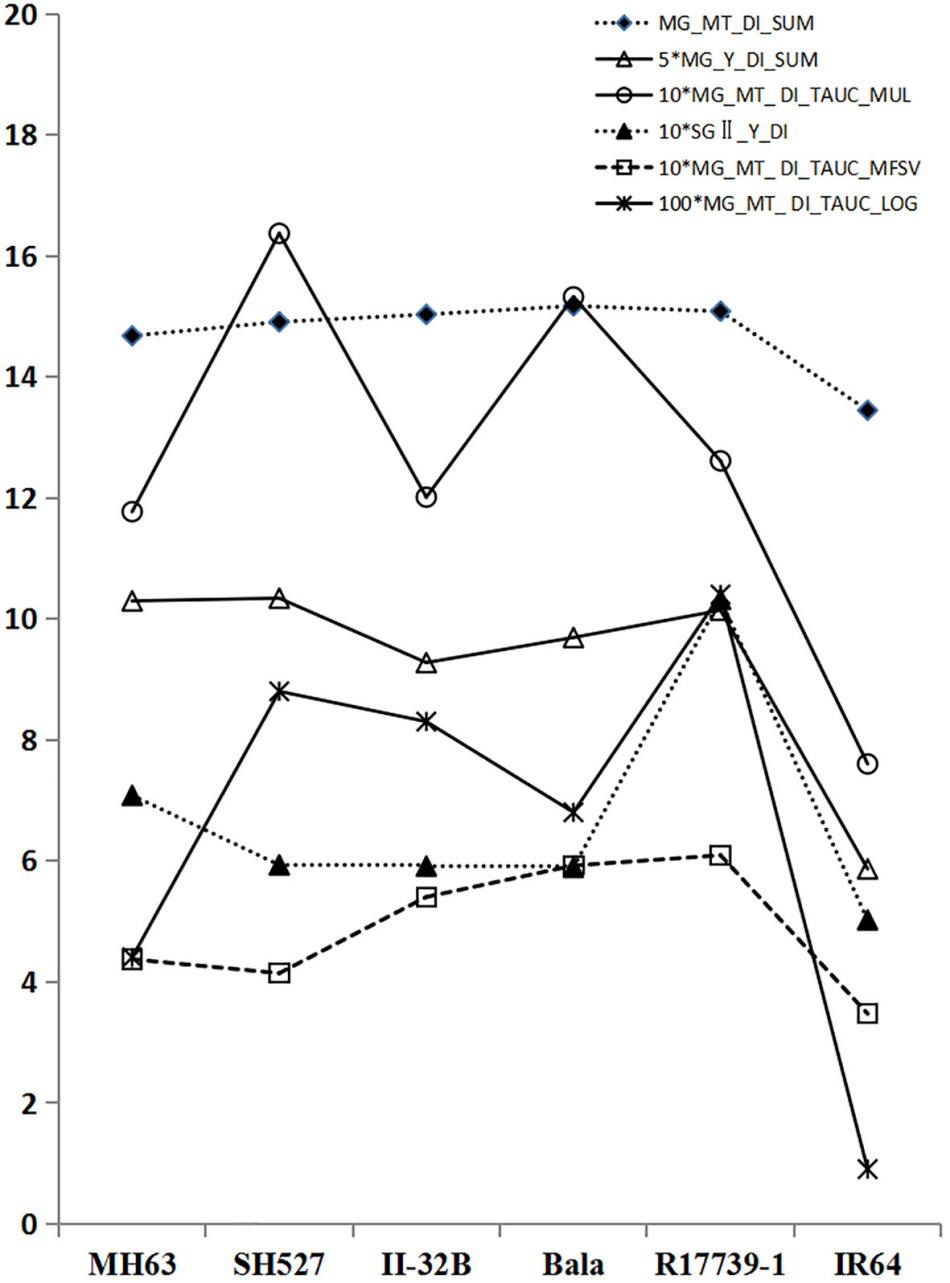
The evaluating effect of six suitable indices.

**Figure 10:**
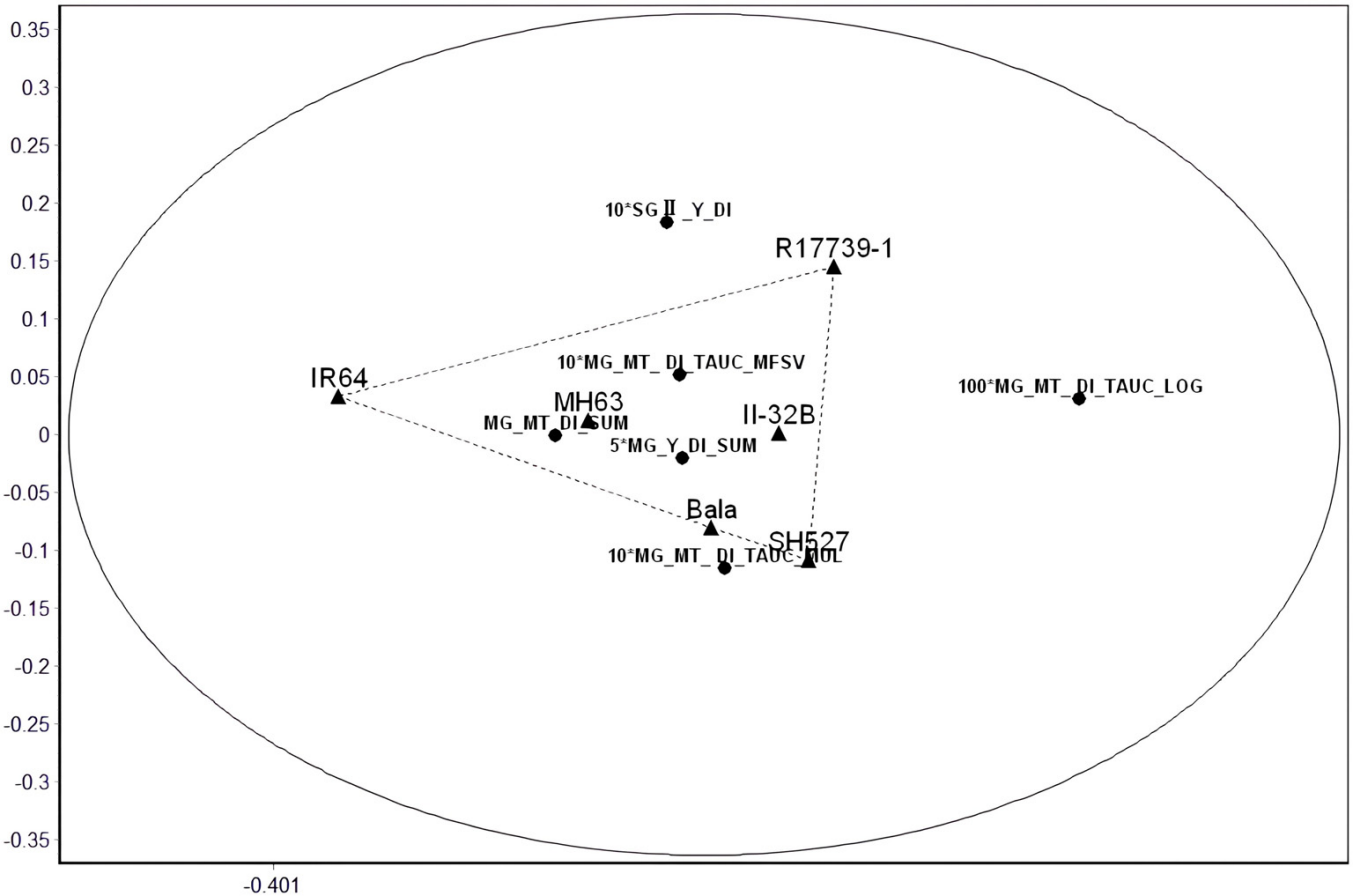
Corresponding analysis chart of six varieties and six suitable indices (ellipse is 95% confidence limit).

The results are shown in **Figure 8**; except for the two indices with all 0 values, the remaining 26 indicators were clustered into four categories, which had different loads in the space of the four principal factors. According to the literature reports and practical experience (refer to Section 2.1 introduction to the test materials), the indicators inconsistent with the drought resistance experience were removed after careful comparison. Six suitable indicators were screened out: multi-gradient multi-trait drought resistance index and (MG_MT_DI_SUM), multi-gradient yield drought resistance index and (MG_Y_DI_SUM), multi-gradient and multi-trait drought resistance index map of the total area (MG_MT_DI_TAUC_MUL), gradient secondary yield drought resistance index (SGII._Y_DI), and the composite value of the membership function of the total area of the multi-gradient and multi-trait drought resistance index map (MG_MT_DI_TAUC_MFSV) and the logarithm of the total area of the multi-gradient and multi-trait drought resistance index map (MG_MT_DI_TAUC_LOG). The evaluation effects of the last five indicators were compared on a single graph after magnifying them by 5, 10, 10, 10, and 100 times (**Figure 9**). The corresponding analysis results of the six tested varieties and these six indices are shown in **Figure 10**. It is obvious that relative to IR64, R17739-1 and SH527 are the two most important factors in drought resistance. From the perspective of the ability of indicators to distinguish the drought resistance of varieties (**Figure 9**), four indicators, namely, 100*MG_MT_DI_TAUC_LOG, 10*MG_MT_DI_TAUC_MUL, 5*MG_Y_DI_SUM, and 10*SGII._Y_DI, can clearly distinguish the comprehensive drought resistance of the six tested varieties. Combined with **Figures 9** and **10**, it can be considered that 100*MG_MT_DI_TAUC_LOG and 5*MG_Y_DI_SUM are ideal evaluation indicators.

### 3.7 Drought resistance evaluation of varieties

**Figure 11:**
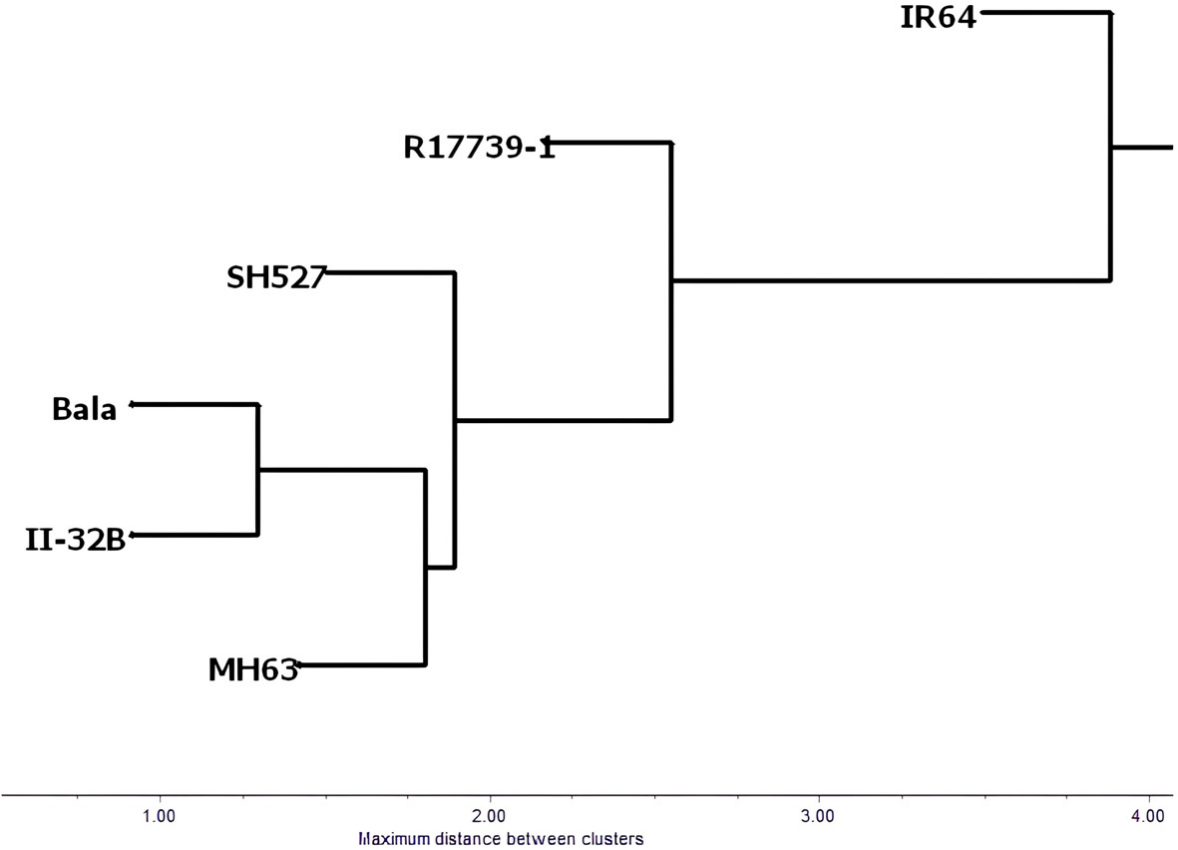
Clustering of six varieties with six suitable indices.

Six composite evaluation indices of drought resistance with good effects, namely, MG_MT_DI_SUM, 5*MG_Y_DI_SUM, 10*MG_MT_DI_TAUC_MUL, 10*SGII._Y_DI, 10*MG_MT_DI_TAUC_MFSV, and 100*MG_MT_DI_TAUC_LOG, were selected to systematically cluster and analyze the drought resistance of six varieties (materials). The results are shown in **Figure 11**, combined with **Figures 9** and **10**. It can be seen that the drought resistance of the six tested varieties is in the following order: R17739-1 > SH527 > MH6 3 > Bala ≧ II.-32B > IR64.

## 4 Discussion

### 4.1 Selection of drought resistance test method

The drought resistance of rice depends on the interaction between heredity and the environment. Only when the environmental conditions are consistent and repeatable can the drought resistance of rice varieties be correctly compared. For drought resistance, it is mainly soil moisture content. However, the dynamic change and uncertainty of soil water content often make the drought resistance tests challenging to repeat, and the results of multiple tests may be inconsistent (Rawat et al., 2020). This makes it very difficult to identify rice drought resistance. For the results to be consistent and repeatable, soil moisture content must be precisely controlled (Bogati and Walczak, 2022). More attention has been paid to monitoring soil moisture content. It has been reported that soil water control treatments such as 30% soil volumetric water content ~ saturated water content, (75% ± 5%) field water capacity, and 70% maximum field water capacity have been used to identify drought resistance of rice. Some use 60% and 70% saturated water content as the lower irrigation limit in each growth period (Kumari et al., 2022). The effects of sustained moderate stress on rice yield and WUE were studied. Shanghai Agricultural Biological Gene Center has established an identification facility “based on soil water transitivity”, which can realize irrigation treatment with different transitivity for the same genotype in the same field. This method has a gradient system but cannot quantify soil water control. In this paper, a wide water ecological gradient quantitative water control test was designed, ranging from saturated water content to 40% FMC. The four designed quantitative water control treatments formed four water gradients with very significant differences. They had a very substantial impact on the yield structure WUE and other characteristics of the tested varieties. The test process clearly and finely controlled the soil water content. The results are stable and repeatable, which is an ideal test method. Due to the different tolerance degrees of varying rice varieties to drought stress, some may have exceeded the tolerance limit under a single drought stress, and some are still within the tolerance range. Therefore, a single drought stress gradient cannot accurately evaluate drought resistance, and multi-gradient comprehensive evaluation is a more reasonable method.

### 4.2 Algorithm problem in drought resistance evaluation

The early drought resistance evaluation algorithms are mainly developed from the concepts of DC and DRI. In the research on drought resistance evaluation, it was found that these two algorithms have certain limitations. Ji Tianhui et al. believed that the drought resistance coefficient only represented the sensitivity of varieties to drought, reflected the stable yield of varieties but did not reflect the yield level, and was not suitable for selecting varieties aiming at harvesting economic yield. Lan Jusheng proposed the drought resistance index DRI based on an improved drought resistance coefficient. The product of drought resistance coefficient DC (Yd/Yp) reflecting the interaction effect between genotype and environment and yield potential (Yd/Ymp) under water stress was taken as DRI, which was closer to the actual drought resistance of test varieties. The author agrees with the views of Hu Biaolin and Lan Jusheng et al. that DRI is more scientific and appropriate than DC. Hu Fushun proposed the DI algorithm, introduced the control variety as a reference in DRI, and determined the drought resistance level of the tested variety by comparing it with the control variety. Ji Tianhui et al. believed that DI was a comprehensive drought resistance identification index most suitable for drought resistance breeding and regional tests, taking the performance of control varieties as a reference and taking into account the relative yield (DC) and absolute yield of varieties, which was convenient for combination with variety district test and variety yield comparison test. Zheng Guiping proposed expanding the “drought resistance index” to the “comprehensive drought resistance index”, aiming at the comprehensive drought resistance index for evaluating crop yield and quality. Recently, much literature has reported that D-value algorithms use membership function values. The membership function value algorithm is obtained by dividing the difference between the DI or DC of a particular trait and the minimum DI or DC of the trait in all varieties by the difference between the maximum and minimum DI or DC of the trait. The D-value algorithm is based on the DC value of the ith trait of each strain and its maximum and minimum values of DC in all the tested strains to calculate the membership function value μ(xi) of the trait, then calculate the weight value ri of each trait in all the drought resistance indicators using the DC value, and then calculate the product sum of μ(xi) and ri of all the selected traits of each strain. The D value was used to evaluate the comprehensive drought resistance of each strain. Using the membership function value to calculate the integrated drought resistance (D-value) algorithm is a great advance. Still, because DI is more scientific and suitable than DC, it is better to use the D-value algorithm based on DI to comprehensively and systematically evaluate rice’s drought resistance. There are also membership function algorithms, comprehensive membership function value, membership function and principal component analysis, membership function combined with drought resistance index, membership function combined with GGE biplot DI algorithm, etc. Statistical analysis algorithms include factor analysis, principal component analysis, gray correlation analysis, correlation analysis, gray correlation, stepwise regression, cluster analysis, and algorithms that combine statistical analysis with DC and DI. These algorithms have innovated and developed crop drought resistance evaluation techniques. However, different algorithms have obtained different evaluation results, bringing much uncertainty to the drought resistance evaluation work. Hence, comparing many algorithms is significant in researching drought resistance evaluation algorithms.

A new algorithm is proposed in this paper to address the problem of comprehensive evaluation of drought resistance under gradient quantitative water control. It is adapted to all indices and all drought stress gradients using the yield drought resistance index. The closed graph area (TAUC) of each DI point and horizontal axis in the graph was calculated, regarded as the total effect of yield and other traits of each variety/material on the change in soil water condition under the condition of gradient quantified water control. On this basis, the sum, product, logarithm, and MFSV values of TAUC for each trait were calculated, which were used as the comprehensive effect values of multi-gradient and multi-trait water ecology of rice varieties/materials tested. The area algorithm based on DI value is used to realize the unified and comprehensive comparison of water saving and drought resistance under multiple gradients, and the evaluation problem caused by different identification results of drought resistance under different gradients was solved. At the same time, a logarithmic index algorithm based on the DI value area was proposed. Compared with more than 20 kinds of drought resistance evaluation indices calculated by combining D-value and membership function algorithms, the actual evaluation effect has certain advantages.

### 4.3 Selection of drought resistance evaluation index

The ultimate goal of drought resistance evaluation is to use some evaluation indices to identify the drought resistance of varieties (Kumar et al., 2017). Through the calculation and in-depth comparative analysis of 28 evaluation indices of six test varieties (**Table 1**, **Figures 7**-**10**), it can be seen that in terms of evaluation rationality, the yield drought resistance coefficient is not as good as the yield drought resistance index. For example, IR64 is a more recognized drought-sensitive variety, and the results ranked by drought resistance index under 80% FMC are more realistic (Mishra et al., 2019). However, using the drought resistance coefficient to sort the results is impractical. Under different gradients with significant differences, the ranking results of drought resistance of the six rice varieties were very inconsistent. For example, the ranking results of IR64 were significantly different under mild and severe stress. Therefore, it is difficult to evaluate drought resistance under single-gradient drought stress correctly, and the evaluation effect of the multi-gradient evaluation index is more reasonable than that of the single gradient. However, the correlation between the yield drought resistance coefficient of gradient II and III (Sii_Y_DC and Siii_Y_DC), the drought resistance index of gradient 3 and (Siii_MT_DI_SUM), and the multi-gradient indices showed that gradients 2 and 3 had an obvious influence on the multi-gradient indices. That is to say, the difference in drought resistance revealed by the multi-gradient indicators mainly stems from the difference in performance of gradients 2 and 3.

Among the six preliminary screening indicators [i) MG_MT_DI_SUM, ii) MG_Y_DI_SUM, iii) MG_MT_DI_TAUC_MUL, iv) SG-II_Y_DI, v) MG_MT_DI_TAUC_MFSV, and vi) MG_MT_DI_TAUC_LOG)], MG_MT_DI_SUM is a common factor with large loads on the four main factor vectors among the 26-factor analysis indicators. It is the main index with the most obvious comprehensive change in variety drought resistance, which can reflect the comprehensive difference of multi-trait drought resistance of various varieties. However, the discrimination ability of MG_MT_DI_SUM among the six test varieties is weak, and it is not ideal as an evaluation index. The corresponding analysis diagram reflects the relative magnitude of drought resistance of each array on the first and second principal component factor vectors, indicating that the drought resistance of R17739-1 and SH527 is due to the other four varieties. After the unsuitable indicators are removed from the comparison with **Figure 10**, two indicators remain: MG_Y_DI_SUM and MG_MT_DI_TAUC_LOG. If the discrimination between varieties is considered, 100*MG_MT_DI_TAUC_LOG is an ideal evaluation index. The biological significance of MG_MT_DI_TAUC_LOG is the comprehensive response of yield and its constituent characteristics, biological WUE, and grain WUE to soil water content, and its theoretical value is between 0 and 16.

Many evaluation indices of drought resistance and selection traits of drought-resistant breeding have been reported in the literature, such as the drought stress index based on seed setting rate (Singh and Laxmi, 2015), water stress index WSI based on seed setting rate, drought tolerance index or drought tolerance index, and composite evaluation system based on comprehensive evaluation index D and drought tolerance index DI. The extensive drought resistance index K value was constructed based on relative plant height, seed setting rate, and the number of panicle days, and the comprehensive drought tolerance index comprised of sowing date, plant height, and panicle weight; plant height and panicle length were taken as the identification indices of drought resistance, and effective panicle number, number of grains per panicle, ear neck thickness, grain width, etc., were used. Grain width, ear neck thickness, number of grains per ear, effective ear per plant, and seed setting rate were used as comprehensive evaluation indices. In this paper, yield and its component traits (GYP, PPP, FGP, and TGW) and water use efficiency (GWUE and BWUE) were mainly taken as key traits, and drought resistance evaluation of gradient quantitative water control experiment was carried out under a wide water ecological range, which is different from previous studies. The results obtained in this way are more reliable.

## Data Availability

The original contributions presented in the study are included in the article/supplementary material. Further inquiries can be directed to the corresponding author.

## References

[B1] Alexandrov N. Tai S. Wang W. Mansueto L. Palis K. Fuentes R. R.. (2015). SNP-Seek database of SNPs derived from 3000 rice genomes. Nucleic Acids Res. 43, D1023–D1027. doi: 10.1093/NAR/GKU1039 25429973 PMC4383887

[B2] Ali J. Xu J. L. Gao Y. M. Ma X. F. Meng L. J. Wang Y.. (2017). Harnessing the hidden genetic diversity for improving multiple abiotic stress tolerance in rice (Oryza sativa L.). PloS One 12, e0172515. doi: 10.1371/JOURNAL.PONE.0172515 28278154 PMC5344367

[B3] Anjum S. A. Ashraf U. Tanveer M. Khan I. Hussain S. Shahzad B.. (2017). Drought induced changes in growth, osmolyte accumulation and antioxidant metabolism of three maize hybrids. Front. Plant Sci. 8. doi: 10.3389/FPLS.2017.00069 PMC5292435 28220130

[B4] Anjum S. Xie X. Wang L. Saleem M. Man C. Lei W. (2011). Morphological, physiological and biochemical responses of plants to drought stress. Afr. J. Agric. Res. 6 (9), 2026–2032. doi: 10.5897/AJAR10.027

[B5] Aroca R. (2013). Plant Responses to Drought Stress: From Morphological to Molecular Features. (Heidelberg, Germany: Springer), 1–466. doi: 10.1007/978-3-642-32653-0/COVER

[B6] Augustine R. C. Vierstra R. D. (2018). SUMOylation: re-wiring the plant nucleus during stress and development. Curr. Opin. Plant Biol. 45, 143–154. doi: 10.1016/J.PBI.2018.06.006 30014889

[B7] Auler P. A. do Amaral M. N. Rodrigues G. S. Benitez L. C. da Maia L. C. Souza G. M.. (2017). Molecular responses to recurrent drought in two contrasting rice genotypes. Planta 246, 899–914. doi: 10.1007/S00425-017-2736-2/FIGURES/11 28702689

[B8] Bailey-Serres J. Parker J. E. Ainsworth E. A. Oldroyd G. E. D. Schroeder J. I. (2019). Genetic strategies for improving crop yields. Nature 575, 109–118. doi: 10.1038/S41586-019-1679-0 31695205 PMC7024682

[B9] Barik S. R. Pandit E. Pradhan S. K. Mohanty S. P. Mohapatra T. (2019). Genetic mapping of morpho-physiological traits involved during reproductive stage drought tolerance in rice. PloS One 14, e0214979. doi: 10.1371/JOURNAL.PONE.0214979 31846460 PMC6917300

[B10] Basu S. Jongerden J. Ruivenkamp G. (2017). Development of the drought tolerant variety Sahbhagi Dhan: exploring the concepts commons and community building. Int. J. Commons 11, 144–170. doi: 10.18352/IJC.673

[B11] Beznec A. Faccio P. Miralles D. J. Abeledo L. G. Oneto C. D. Garibotto M.. (2021). Stress-induced expression of IPT gene in transgenic wheat reduces grain yield penalty under drought. J. Genet. Eng. Biotechnol. 19, 1–17. doi: 10.1186/S43141-021-00171-W PMC8110665 33970377

[B12] Bhatnagar N. Kim R. Han S. Song J. Lee G. S. Lee S.. (2020). Ectopic expression of osPYL/RCAR7, an ABA receptor having low signaling activity, improves drought tolerance without growth defects in rice. Int. J. Mol. Sci. 21, 4163. doi: 10.3390/IJMS21114163 32545174 PMC7312952

[B13] Bi J. Hou D. Zhang X. Tan J. Bi Q. Zhang K.. (2021). A novel water-saving and drought-resistance rice variety promotes phosphorus absorption through root secreting organic acid compounds to stabilize yield under water-saving condition. J. Clean Prod 315, 127992. doi: 10.1016/J.JCLEPRO.2021.127992

[B14] Bogati K. Walczak M. (2022). The impact of drought stress on soil microbial community, enzyme activities and plants. Agronomy 12, 189. doi: 10.3390/AGRONOMY12010189

[B15] Chen X. Ding Y. Yang Y. Song C. Wang B. Yang S.. (2021). Protein kinases in plant responses to drought, salt, and cold stress. J. Integr. Plant Biol. 63, 53–78. doi: 10.1111/JIPB.13061 33399265

[B16] Chengqi Z. Yuxuan Y. Tian Q. Yafan H. Jifeng Y. Zhicheng S. (2024). Drought-tolerant rice at molecular breeding eras: an emerging reality. Rice Sci. 31, 179–189. doi: 10.1016/J.RSCI.2023.11.005

[B17] Choudhury D. Mukherjee C. Dey S. Dutta S. (2024). Drought stress tolerance in rice: a critical insight. Plant Sci. Today 11, 241–257. doi: 10.14719/PST.2613

[B18] Chukwu S. C. Rafii M. Y. Ramlee S. I. Ismail S. I. Oladosu Y. Okporie E.. (2019). Marker-assisted selection and gene pyramiding for resistance to bacterial leaf blight disease of rice (Oryza sativa L.). Biotechnol. Biotechnol. Equip. 33, 440–455. doi: 10.1080/13102818.2019.1584054

[B19] Cui Y. Zhang W. Lin X. Xu S. Xu J. Li Z. (2018). Simultaneous improvement and genetic dissection of drought tolerance using selected breeding populations of rice. Front. Plant Sci. 9. doi: 10.3389/FPLS.2018.00320/BIBTEX PMC5862857 29599789

[B20] Dixit S. Singh A. Sandhu N. Bhandari A. Vikram P. Kumar A. (2017). Combining drought and submergence tolerance in rice: marker-assisted breeding and QTL combination effects. Mol. Breed. 37, 1–12. doi: 10.1007/S11032-017-0737-2/TABLES/3 29151804 PMC5670188

[B21] Efendi Bakhtiar Zakaria S. Hakim L. Sobrizal (2017). Mutation With Gamma Raysirradiation to Assemble Green Super Rice Tolerant to Drought Stress and high Yield Rice (Oryza Sativa l.). Int. J. Adv. Science Eng. Technology(IJASEAT) 5, 1–5. doi: JASEAT-IRAJ-DOIONLINE-9050

[B22] Fahad S. Bajwa A. A. Nazir U. Anjum S. A. Farooq A. Zohaib A.. (2017). Crop production under drought and heat stress: plant responses and management options. Front. Plant Sci. 8. doi: 10.3389/FPLS.2017.01147 PMC5489704 28706531

[B23] Fukagawa N. K. Ziska L. H. (2019). Rice: importance for global nutrition. J. Nutr. Sci. Vitaminol (Tokyo) 65, S2–S3. doi: 10.3177/JNSV.65.S2 31619630

[B24] Gopi G. Manjula M. (2018). Speciality rice biodiversity of Kerala: Need for incentivizing conservation in the era of changing climate. Curr. Sci. 114, 997–1006. doi: 10.18520/CS/V114/I05/997-1006

[B25] Gupta A. Rico-Medina A. Caño-Delgado A. I. (2020). The physiology of plant responses to drought. Sci. (1979) 368, 266–269. doi: 10.1126/SCIENCE.AAZ7614 32299946

[B26] Han J. Singh V. P. (2023). A review of widely used drought indices and the challenges of drought assessment under climate change. Environ. Monit. Assess. 195, 1438. doi: 10.1007/s10661-023-12062-3 37943470

[B27] He Z. Zhang P. Jia H. Zhang S. Nishawy E. Sun X.. (2024). Regulatory mechanisms and breeding strategies for crop drought resistance. New Crops 1, 100029. doi: 10.1016/J.NCROPS.2024.100029

[B28] Hussain H. A. Hussain S. Khaliq A. Ashraf U. Anjum S. A. Men S.. (2018). Chilling and drought stresses in crop plants: Implications, cross talk, and potential management opportunities. Front. Plant Sci. 9. doi: 10.3389/FPLS.2018.00393/BIBTEX PMC5902779 29692787

[B29] Khanna A. Anumalla M. Catolos M. Bartholomé J. Fritsche-Neto R. Platten J. D.. (2022). Genetic trends estimation in IRRIs rice drought breeding program and identification of high yielding drought-tolerant lines. Rice (N Y) 15, 1–14. doi: 10.1186/S12284-022-00559-3 PMC8898209 35247120

[B30] Kim Y. Chung Y. S. Lee E. Tripathi P. Heo S. Kim K. H. (2020). Root response to drought stress in rice (Oryza sativa L.). Int. J. Mol. Sci. 21, 1–22. doi: 10.3390/IJMS21041513 PMC7073213 32098434

[B31] Kumar A. Basu S. Ramegowda V. Pereira A. (2017). Mechanisms of drought tolerance in rice. (Sawston, Cambridge UK: Burleigh Dodds Science Publishing Limited), 131–163. doi: 10.19103/AS.2106.0003.08

[B32] Kumar M. Patel M. K. Kumar N. Bajpai A. B. Siddique K. H. M. (2021). Metabolomics and molecular approaches reveal drought stress tolerance in plants. Int. J. Mol. Sci. 22, 1–23. doi: 10.3390/IJMS22179108 PMC8431676 34502020

[B33] Kumari V. V. Banerjee P. Verma V. C. Sukumaran S. Chandran M. A. S. Gopinath K. A.. (2022). Plant nutrition: an effective way to alleviate abiotic stress in agricultural crops. Int. J. Mol. Sci. 23, 1–30. doi: 10.3390/IJMS23158519 PMC9368943 35955651

[B34] Luo L. J. (2010). Breeding for water-saving and drought-resistance rice (WDR) in China. J. Exp. Bot. 61, 3509–3517. doi: 10.1093/JXB/ERQ185 20603281

[B35] Luo L. Mei H. Yu X. Xia H. Chen L. Liu H.. (2019). ater-saving and drought-resistance rice: from the concept to practice and theory. Mol. Breed. 39, 1–15. doi: 10.1007/S11032-019-1057-5/FIGURES/5

[B36] Manickavelu A. Nadarajan N. Ganesh S. K. Gnanamalar R. P. Chandra Babu R. (2006). Drought tolerance in rice: Morphological and molecular genetic consideration. Plant Growth Regul. 50, 121–138. doi: 10.1007/S10725-006-9109-3/FIGURES/2

[B37] Martos V. Ahmad A. Cartujo P. Ordoñez J. (2021). Ensuring agricultural sustainability through remote sensing in the era of agriculture 5.0. Appl. Sci. 11, 5911. doi: 10.3390/APP11135911

[B38] Mishra S. S. Behera P. K. Panda D. (2019). Genotypic variability for drought tolerance-related morpho-physiological traits among indigenous rice landraces of Jeypore tract of Odisha, India. J. Crop Improv 33, 254–278. doi: 10.1080/15427528.2019.1579138

[B39] Mukherjee S. Mishra A. Trenberth K. E. (2018). Climate change and drought: a perspective on drought indices. Curr. Clim Change Rep. 4, 145–163. doi: 10.1007/s40641-018-0098-x

[B40] Ortega‐Gaucin D. Ceballos‐tavares J. A. Sánchez A. O. Castellano‐bahena H. V. (2021). Agricultural drought risk assessment: A spatial analysis of hazard, exposure, and vulnerability in Zacatecas, Mexico. Water (Switzerland) 13, 1431. doi: 10.3390/W13101431/S1

[B41] Panda D. Mishra S. S. Behera P. K. (2021). Drought tolerance in rice: focus on recent mechanisms and approaches. Rice Sci. 28, 119–132. doi: 10.1016/J.RSCI.2021.01.002

[B42] Pang Y. Chen K. Wang X. Xu J. Ali J. Li Z. (2017). Recurrent selection breeding by dominant male sterility for multiple abiotic stresses tolerant rice cultivars. Euphytica 213, 1–13. doi: 10.1007/S10681-017-2055-5/TABLES/4 PMC6956909 31997828

[B43] Rawat M. Arunachalam K. Arunachalam A. Alatalo J. M. Kumar J. Simon B.. (2020). Relative contribution of plant traits and soil properties to the functioning of a temperate forest ecosystem in the Indian Himalayas. CATENA 194 (1), 104671. doi: 10.1016/j.catena.2020.104671

[B44] Raza A. Mubarik M. S. Sharif R. Habib M. Jabeen W. Zhang C.. (2023). Developing drought-smart, ready-to-grow future crops. Plant Genome 16, 1–37. doi: 10.1002/TPG2.20279 PMC12807413 36366733

[B45] Salehi-Lisar S. Y. Motafakkerazad R. M. M. Rahm M. I. M. (2012). “Water stress in plants: causes, effects and responses,” in Water Stress (London, UK: IntechOpen Limited). doi: 10.5772/39363

[B46] Seleiman M. F. Al-Suhaibani N. Ali N. Akmal M. Alotaibi M. Refay Y.. (2021). Drought stress impacts on plants and different approaches to alleviate its adverse effects. Plants (Basel) 10, 1–25. doi: 10.3390/PLANTS10020259 PMC7911879 33525688

[B47] Shultana R. Zuan A. T. K. Yusop M. R. Saud H. M. Ayanda A. F. (2020). Effect of salt-tolerant bacterial inoculations on rice seedlings differing in salt-tolerance under saline soil conditions. Agronomy 10, 1030. doi: 10.3390/AGRONOMY10071030

[B48] Singh D. Laxmi A. (2015). Transcriptional regulation of drought response: a tortuous network of transcriptional factors. Front. Plant Sci. 6. doi: 10.3389/FPLS.2015.00895 PMC4625044 26579147

[B49] Singh R. Singh Y. Xalaxo S. Verulkar S. Yadav N. Singh S.. (2016). From QTL to variety-harnessing the benefits of QTLs for drought, flood and salt tolerance in mega rice varieties of India through a multi-institutional network. Plant Sci. 242, 278–287. doi: 10.1016/J.PLANTSCI.2015.08.008 26566845

[B50] Wu G. Zuo X. Wu W. Ren L. Wu C. Lin Y.. (2024). Late Neolithic to Bronze Age water management and upland rice cultivation in the mountainous areas of Southeastern China Coast. Quaternary Int. 680, 55–63. doi: 10.1016/J.QUAINT.2023.11.008

